# Dynamical system with plastic self-organized velocity field as an alternative conceptual model of a cognitive system

**DOI:** 10.1038/s41598-017-16994-y

**Published:** 2017-12-05

**Authors:** Natalia B. Janson, Christopher J. Marsden

**Affiliations:** Department of Mathematical Sciences, Loughborough University, Loughborough, LE11 3TU UK

## Abstract

It is well known that architecturally the brain is a neural network, i.e. a collection of many relatively simple units coupled flexibly. However, it has been unclear how the possession of this architecture enables higher-level cognitive functions, which are unique to the brain. Here, we consider the brain from the viewpoint of dynamical systems theory and hypothesize that the unique feature of the brain, the self-organized plasticity of its architecture, could represent the means of enabling the self-organized plasticity of its velocity vector field. We propose that, conceptually, the principle of cognition could amount to the existence of appropriate rules governing self-organization of the velocity field of a dynamical system with an appropriate account of stimuli. To support this hypothesis, we propose a simple non-neuromorphic mathematical model with a plastic self-organized velocity field, which has no prototype in physical world. This system is shown to be capable of basic cognition, which is illustrated numerically and with musical data. Our conceptual model could provide an additional insight into the working principles of the brain. Moreover, hardware implementations of plastic velocity fields self-organizing according to various rules could pave the way to creating artificial intelligence of a novel type.

## Introduction

Two most popular and successful paradigms for a cognitive system are that of a computer and of a neural network. The computer paradigm is based on a conceptual model of human thinking as a process of going from one statement to another according to a prescribed set of rules (an algorithm), and does not take into account any knowledge about the physical architecture of the natural cognitive system–the brain. On the contrary, the neural network paradigm is based on the knowledge of the physical structure of the natural cognitive device–the brain–as primarily a collection of communicating units. However, how this architecture gives rise to cognition is not properly understood at a conceptual level^1^. Since in most practical applications neural networks are simulated on a computer, many modern artificial neural networks represent a combination of the two paradigms.

One of the major challenges of modern science is to understand the brain. Despite impressive achievements both in artificial intelligence and in biological neuroscience, the question about the way cognitive functions arise from the brain architecture remains open^1–5^, and to date there exists no definitive conceptual model of the brain^6^. Given the similarity in functions, a computer metaphor has been widely used to describe the brain function, but has not delivered the insights required because of the architectural differences between the two devices^7^. In the last few decades there has been a lot of effort trying to understand the brain through the prism of the dynamical systems theory. This approach largely amounted to reproducing in a dynamical model the physical architecture of the brain as a neural network, in which physical and chemical mechanisms acting within individual cells and at the points of their interaction have been described with various degrees of detalisation using experimentally available brain data^8,9^. It has been hoped that incorporating more details in a model would somehow provide more conceptual insight into what the brain does^1^. However, in the absence of a decisive breakthrough to date, this approach has started to raise concerns, because it has been unclear what level of detail should be used for the brain simulation and what questions to ask of the brain data^10,11^. Quoting from^3^, “It is a chicken and egg situation: Once we know how the brain works, we’ll know how to look at the data”.

In parallel, there have been several attempts to model cognitive processes phenomenologically, i.e. without linking them to the physical processes in the brain, based on an assumption that a cognitive system was formed by some cognitive agents represented as dynamical systems^12,13^. However, these have not yet resulted in a breakthrough in our understanding of cognition. Moreover, it has been suggested that the theory of dynamical systems itself has not been ready to form the foundation for cognitive science and needed extensions directly relevant to cognitive processing^14^, without specifying exactly what concepts it was lacking and what sort of extentions it needed.

Here, we adopt an approach *opposite* to detailed brain modelling, but inspired by the analysis of the brain models available. Namely, assuming that the brain is in principle describable as a dynamical system of the form (7), we attempt to see through the intricacies of numerous mechanisms operating in the brain and to identiy an object, which could shed the light on the conceptual idea behind the brain organization. We propose that this candidate object is the velocity vector field of the brain, which has not been in the focus of brain research previously. The velocity field is a critical element of a dynamical model, and is a mathematical embodiment of a ruling force that dictates how the system should behave in every feasible situation. In an accurate realistic model of the brain, the velocity field would directly instruct every neuron when to fire. By looking at the conventional brain models, we figure out that thanks to the brain plasticity, i.e. to the perpetual spontaneous modifications of the brain parameters, such as connections^15^ and neural excitability^16^, the brain’s velocity field continually evolves. After that we take a conceptual leap and hypothesize that, given the traditionally assumed existence of deterministic laws underlying the brain plasticity^17^, there could also exist *deterministic* laws governing spontaneous evolution, self-organization, of the brain’s velocity field. We further hypothesize that this self-organized plasticity of the brain’s velocity field could be the factor enabling cognition, and that the laws governing the evolution of the brain’s connections provide the mechanism by which the brain’s velocity field can evolve according to laws suitable for cognition. For the brain, the existence of deterministic laws governing evolution of the velocity field would need to be verified, and although seems plausible, is not immediately obvious at this stage. However, we take another step forward and hypothesize that, regardless of whether this principle does operate in the brain, it could still underlie cognition. Namely, cognition could be associated with the existence of in-built appropriate rules governing spontaneous evolution of the velocity field of a general dynamical system being affected by stimuli. With this, such a system does not need to be a neural network. We thus formulate an alternative conceptual model of a cognitive system as a dynamical system with plastic self-organizing velocity field expressed by (7)–(8).

Self-organizing velocity fields have not been considered within the theory of dynamical systems to date, so in order to be able to formalize and verify our hypothesis, we introduce such fields into consideration. We construct an example (9), (14) of a non-neuromorphic dynamical system, which processes stimuli by self-organizing its velocity field according to some very simple rules, and demonstrate how it performs basic cognition. We thus support our hypothesis about the possible principles underlying cognition. On the way to formulating our conceptual model, we need to find some version of a solution to the long-standing problem of memory representation in the brain. By putting together the existing ideas about memory representation from biological neuroscience and from computational neuroscience, and by considering them next to the concept of the velocity field from mathematics, we point out that each of them represents a certain manifestation, and can be explained in terms, of the same velocity field of the brain. We thus put forward a hypothesis that the whole of the memory of a cognitive system is embodied in its velocity field, and that separate elements of this field represent different individual memories. We support this idea by the demonstration of the performance of our simple model (9), (14).

Congitive functions have well been appreciated to represent an *emergent* property of the brain, i.e. the property which arises from interactions between the brain components, and which cannot be reduced to the sum of their individual properties^4,18–20^. However, to date this emergent property has not been identified with any specific experimentally measurable object^20,21^ and thus evaded direct studies. We point out that the brain’s velocity field meets the criteria for an emergent property of the brain and, moreover, its approximations could be potentially reconstructed from detailed brain measurements. This serves as an additional argument towards associating the brain’s velocity field with cognition.

In “Methods and Results” we introduce our conceptual model of a cognitive system and validate it by constructing a simple example of this model and by demonstrating how it processes stimuli. In “Discussion” we discuss the potential impact of individual components of this model on neuroscience and on artificial intelligence. We also briefly comment about the possibility to obtain the velocity field of the brain from detailed brain measurements, and about the possible approach to verify our brain hypothesis.

## Methods and Results

### Velocity vector field

Given the centrality of the velocity vector field to our theory, in this Section we briefly revise its definition, key features and relevance to the underlying physical system.

The working principles of many man-made devices are based on their ability to evolve *spontaneously*. Examples include a pendulum clock^22^ and a vacuum-tube circuit generating electromagnetic oscillations^23^. In addition, in the 20th century the dynamical nature of *living* systems has been appreciated at the levels of a cell, an organ, an organism, and a population of organisms, meaning that their states are spontaneously evolving. Devices^22^ and organs^24,25^ of this type have been modelled as dynamical systems.

A dynamical system is a mathematical way to decsribe the spontaneous evolution of a device and has two ingredients^26^. The first one is a state vector ${\boldsymbol{x}}(t)=({x}_{1}(t),\ldots ,{x}_{N}(t))$, which fully specifies the state of the system at any time *t*, and whose components ${x}_{1},\ldots ,{x}_{N}$ are scalar real quantities representing the state variables that evolve in time. In neurons these are usually membrane voltages, membrane currents, concentrations of various chemicals, etc. As ${x}_{1},\ldots ,{x}_{N}$ are real variables, we have ${\boldsymbol{x}}=({x}_{1},\ldots ,{x}_{N})\in {{\mathbb{R}}}^{N}$, where ${{\mathbb{R}}}^{N}$ is denoted as state space. In other words, the state space is the space of all possible states of the system, and its dimension is equal to the number of state variables *N*. The second ingredient of a dynamical system is a rule that determines how the state evolves with time *t*. For the continuous time and state, the dynamical system is specified by a system of first-order ordinary differential equations (ODEs)

1 $$\frac{{\rm{d}}{x}_{1}}{{\rm{d}}t}={s}_{1}({x}_{1},\ldots ,{x}_{N}),\,\ldots ,\,\frac{{\rm{d}}{x}_{N}}{{\rm{d}}t}={s}_{N}({x}_{1},\ldots ,{x}_{N}),$$

where ${s}_{1},\ldots ,{s}_{N}$ are some functions of the state variables. In a more compact vector notation (1) reads:

2 $$\frac{{\rm{d}}{\boldsymbol{x}}}{{\rm{d}}t}={\boldsymbol{s}}({\boldsymbol{x}}\mathrm{).}$$

**Figure 1:**
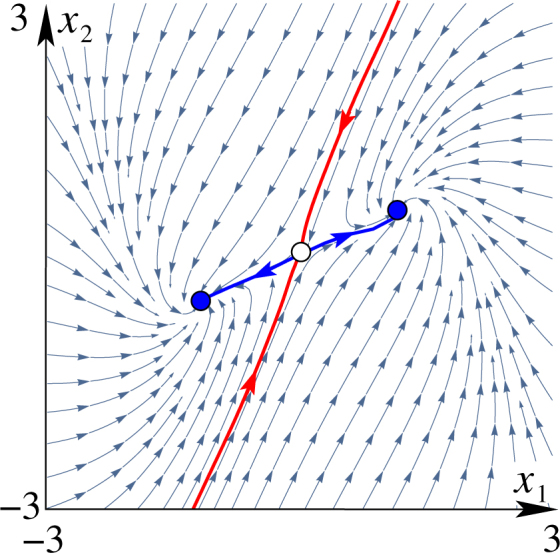
Velocity field of a sample dynamical system. For a two-dimensional dynamical system (3) modelling a neuron, several phase trajectories are shown (thin blue lines) being guided by the velocity vector field (arrows). The features of the velocity field include two stable fixed points (filled circles), the boundary between their basins of attraction (red line), which is a stable manifold of the saddle fixed point (empty circle), and the unstable manifold of the saddle point (thick blue line).

The vector ${\boldsymbol{s}}=({s}_{1},\ldots ,{s}_{N})$ represents the (phase) velocity vector. These vectors are generally different at different points ***x*** in the *N*-dimensional state space, and their full collection is called the *velocity* (vector) *field*. In Fig. 1 this field is shown by arrows for a two-dimensional system being a version of Bonhoeffer-van der Pol model, which is a popular simplified model of a single neuron^27^,

3 $$\frac{{\rm{d}}{x}_{1}}{{\rm{d}}t}={x}_{1}-\frac{{x}_{1}^{3}}{3}-{x}_{2}+I,\,\,\frac{{\rm{d}}{x}_{2}}{{\rm{d}}t}=a(b{x}_{1}-c{x}_{2}),$$

where *I*, *a*, *b* and *c* are some parameters. Here, for illustrative purposes only, we chose the parameter values to be *I* = 0, *a* = 0, *b* = 1 and *c* = 2. Other objects shown in this figure are described in the figure caption. The velocity field specifies in what direction and how fast the state point of the system goes next starting from any given point. It can be loosely understood as a force that governs the behavior of the system by pushing the state ***x*** along a certain trajectory in the state space. When observing the behavior of the system, in a single run of an experiment one registers only a single phase trajectory (or its projection) and therefore only one possible behavioral pattern. However, the velocity field holds information about all possible behavioral patterns the given device can exhibit in principle. Thus, the velocity field is the key ingredient of a dynamical system, because it specifies the *rules* of behavior of the device it describes. In what follows we will consider only smooth systems, such that their velocity fields are differentiable at least twice. Since the final third of the 20th century, biological systems are well appreciated to be both dissipative and non-linear (see^25^ and references therein). For a system to be dissipative means to be loosing energy while functioning. A realistic dynamical model for such a system should be in the form of a dissipative dynamical system, in which the phase volume along the phase trajectories decreases with time.

All spontaneously evolving devices and organs have one feature in common: they possess a well-defined physical architecture. When constructing their models, one applies to their various parts the laws of physics and chemistry formulated as equations, such as algebraic and differential equations, and combines them to obtain one or several differential equations representing the model sought. If the differential equations obtained are ordinary, one rearranges their terms and possibly applies some variable substitutions to obtain a set of first-order ODEs in the form (2). By doing so, one automatically singles out the components of the velocity field ***s*** (see Supplementary Note, Section 1.1., for an illustration of this process). The velocity field of the model (2) can be regarded as a mathematical representation, with a certain degree of accuracy, of the physical structure of the device it describes (see Supplementary Note, page 3, paragraphs 2–3 for explanation). A prominent example is the Hodgkin-Huxley model of a single neuron^28^. Recently, there have been several attempts to model the whole brain as a dynamical system^8,9^. More sophisticated models of physical systems can take the form of delay-differential or partial differential equations (PDEs) to account for spatial effects. However, PDEs can be discretized in space, and delay equations can be discretized with respect to the delay, and either can be ultimately approximated by a set of ODEs of the form (2). Therefore, one can formally speak of the velocity field of an approximate model even in these cases.

One usually assumes that for the given device a perfectly accurate model exists in principle. In practice, the perfect model is unattainable because in the process of modelling less essential details of architecture are usually ignored. However, by including more and more details one approaches the perfect model closer and closer. Mathematically, one can say that the observable behavior of the real device is governed by the velocity field of its unattainable perfect dynamical model. We will refer to this field as the velocity field of the device itself.

Note that the velocity field of any device results from interactions between its components and cannot be explained in terms of the individual properties of these components alone. Therefore, the velocity field satisfies the definition of an *emergent* property of a device, as explained in more detail in Supplementary Note, Section 1. Moreover, an approximation of the velocity field can in principle be obtained from the knowledge of the parameters of the device in the process of creating a dynamical model. Also, segments of this field can be reconstructed from observations of the system behavior as explained in Supplementary Note, Section 2. With this, although the velocity field cannot be measured in an experiment directly, it can be reconstructed from experimental measurements with certain accuracy.

When the physical device is too complex and its realistic modelling is too difficult, equation (2) can be used to describe only a particular phenomenon occurring in it, rather than the device itself. Such models are called phenomenological. An example is Bonhoeffer-van der Pol model^27^, which describes one kind of neural excitability by analogy with a similar phenomenon in an iron wire, and does not show how it is related to the measurable neural variables.

### Conceptual model of a cognitive system

In this Section we analyse the conventional approaches towards the description of the brain as a dynamical system and towards the representation of memories, and introduce an alternative conceptual model of a cognitive system.

#### Modelling the brain: conventional approaches

The brain is known to interact with the environment and to experience time-varying stimuli. In this Section we outline the standard approach to model the brain receiving sensory data, based on non-autonomous dynamical systems.

Stimuli are known to produce one or both of the following effects on the spontaneously evolving devices. Firstly, they can induce time-variation of *parameters* of the system, such as the resting membrane potential of a neuron, which can be modulated by means of transcranial direct current stimulation^29^. Secondly, they can directly modulate the components of the phase *velocity* d***x***/d*t*, such as the time derivative of the neuron’s membrane voltage, which can be amended by applying to the neuron some electric current as described by a model in^30^.

To describe systems interacting with the environment, non-autonomous dynamical systems are used^31^, which differ from (2) in that their velocity fields explicitly depend on time *t*, i.e. $\frac{{\rm{d}}{\boldsymbol{x}}}{{\rm{d}}t}=\tilde{{\boldsymbol{v}}}({\boldsymbol{x}},t)$. Since stimuli are usually represented as time-varying vectors ${\boldsymbol{\eta }}(t)$, the appropriate models take the form^32^

4 $$\frac{{\rm{d}}{\boldsymbol{x}}}{{\rm{d}}t}={\boldsymbol{v}}({\boldsymbol{x}},{\boldsymbol{\eta }}(t\mathrm{)).}$$

If ${\boldsymbol{\eta }}(t)$ is a realization of some random process, systems of the form (4) are called random, or stochastic^33^. The performance of such models can be understood as having every vector of the core velocity field ${\boldsymbol{v}}({\boldsymbol{x}},{\bf{0}})$ (corresponding to the absence of stimulus) forcibly amended at every time *t*. Importantly, after the stimulus ceases (${\boldsymbol{\eta }}(t)={\bf{0}}$), the field ***v*** in (4) instantly regains its core form and keeps no memory of influence it might have experienced previously. With this, the influence of ${\boldsymbol{\eta }}(t)$ on the solution ***x***(*t*) will also disappear, although not instantaneously.

Since the critical cognitive feature is the ability to memorise, in order to describe cognitive systems, the non-autonomous dynamical systems need to be combined in a special way. A conceptual biologically-motivated model of the brain is a neural network, which forms memories as a result of spontaneous unsupervised Hebbian-like learning, which assumes that the strengths of the brain connections should evolve in time according to some deterministic laws^17^. Its mathematical representation is a two-tier combination of equations of the form (4) describing both effects from the stimuli listed above. The first model of this type was proposed in^34^ and can be generalized to the following form:

5 $$\frac{{\rm{d}}{\boldsymbol{x}}}{{\rm{d}}t}={\boldsymbol{a}}({\boldsymbol{x}},{\boldsymbol{w}}(t),{\boldsymbol{\eta }}(t))=\hat{{\boldsymbol{a}}}({\boldsymbol{x}},{\boldsymbol{w}}(t))+{\boldsymbol{\eta }}(t),$$

6 $$\frac{{\rm{d}}{\boldsymbol{w}}}{{\rm{d}}t}={\boldsymbol{b}}({\boldsymbol{w}},{\boldsymbol{x}}(t\mathrm{)).}$$

Here, equation (5) describes evolution of neuron variables ${x}_{1},\ldots ,{x}_{N}$, forming the vector ***x***, in a network of *N* neurons. The neurons are coupled with certain strengths ${w}_{1},\ldots ,{w}_{M}$, forming the vector ***w***, where *M* = *N* ^2^. These strengths vary in time according to (6), and thus continually update the instantaneous shape of the velocity field ***a*** of (5), which ultimately controls the neural behavior. At the same time, the field ***a*** is being directly modulated by the (usually scaled) time-varying external stimulus ${\boldsymbol{\eta }}(t)=({\eta }_{1}(t),\ldots ,{\eta }_{N}(t))$, which both in^34^ and in many other models of neural networks is introduced additively to the core field $\hat{{\boldsymbol{a}}}({\boldsymbol{x}},{\boldsymbol{w}}(t))$.

Note, that in (5) the global velocity field ***a*** of all coupled neurons cannot be represented as a combination of the velocity fields of disconnected individual neurons. Instead, the field acquires its shape as a result of coupling the neurons in a network, as explained in more detail in Supplementary Note, Section 1.2. Therefore, the velocity field satisfies the definition of an emergent property of a neural network.

The version of the model (5)-(6) in^34^ is phenomenological, because every neuron there is represented only by an instantaneous rate of its firings, whose mechanism of occurrence is not included in the model. However, there are many extentions of this model, which use biologically realistic models of the firing neurons and of the spike-timing-dependent-plasticity, e.g. in^35,36^. In models of biological neural networks, functions ***a*** and $\hat{{\boldsymbol{a}}}$ should be nonlinear functions of ***x*** chosen in such a way, that with ${\boldsymbol{\eta }}(t)={\bf{0}}$, at any value of ***w*** the dynamical system (5) describing evolution of ***x*** remains dissipative. Also, in models of biological neural networks, the stimuli affect only a small proportion of neurons, namely, only those which are sensory.

#### Representing memory: unification of conventional approaches

The central concept of neuroscience is neural representation of memories, whose link to the brain architecture remains to be properly understood^10^. There are currently two main paradigms of memory representation. In this Section we interpret them in terms of the dynamical systems theory and show that they can be regarded as different manifestations of the same velocity field of the brain. With this, we suggest that the memory holder in the brain could be the brain’s velocity field. This idea would unify the existing notions about memory storage within a more general approach.

The first memory paradigm states that individual memories are represented as spatio-temporal patterns of activity, or possibly the firing rates, of the relevant neural ensembles^37,38^. Assuming that a certain neural ensemble is described by a dynamical system of the general form (4), its distinct activity patterns are represented as different phase trajectories in the multi-dimensional state space of this system. With this, every phase trajectory is created by the velocity field of the given dynamical model and reveals all the local field’s features, which it encounters on its way. Namely, assuming our system is smooth, the trajectory slows down near the fixed points, spirals around the fixed points of a focus type, follows the manifolds when it approaches them, tends to attractors, and so on. Using the standard approaches of reconstruction of dynamical systems from experimental data^39^, the velocity field can in principle be reconstructed along every trajectory from measurements of all state variables of the given neural ensemble by forming a vector of their time derivatives at every point of the trajectory (see Supplementary Note, Section 2.1.). It is of course well appreciated that any measurements, including the ones of the human brain, can be made with some finite accuracy only, and any parameters or variables recorded as functions of time can be registered only as data discretized in time with some finite sampling frequency, which should comply with Nyquist-Shannon sampling theorem^40^. As a result, the velocity field could be reconstructed from the neural activity data only with some mistake, just like any other quantity estimated from experimental measurements of other quantities. Despite the estimation error, it is still possible to get an idea of how the field is shaped close to the trajectory if all the state variables are available. This memory paradigm can therefore be interpreted as a single memory being represented by a certain local feature of the velocity field of the dynamical model describing the given neural circuit. Then the expression “memory trace” acquires the meaning of a trace on the velocity field of the dynamical system.

Within the second paradigm, memories are stored in the strengths of inter-neuron couplings^41,42^, namely, to hold a certain memory, certain connections should take certain values. A variation of this theory suggests that memories can also be held in voltage-dependent membrane conductances of neurons^16^. Here, we point out that together with the physical architectures of individual neurons and of their circuits, the values of couplings and of mebrane conductances determine the shape of the velocity field that governs the behavior of neural variables. It is this field, which for any initial condition determines what pattern of activity is realized, i.e. what memory is evoked within the first paradigm. The latter observation connects the second memory paradigm with the first one.

Namely, the two memory paradigms can be unified by pointing out that the assignment of certain values to synaptic couplings and membrane conductances is the means to create in the velocity field the required set of features, including those giving rise to the required attractors or manifolds. With this, any given temporal pattern of neural activity is a manifestation of the presence of certain features in the velocity field of the neural network. Here we propose that, assuming that the brain can be described as a dynamical system, individual memories are represented as various elements of its velocity field.

Both the conventional memory paradigms suggest that memories are stored somewhere in the *physical* space of the brain. However, if one regards memories as features of the velocity field, then as such they can only exist in the *non-physical* multi-dimensional state space of the brain. This is consistent with, and constitutes a generalization from, both a statement in^43^ that word representations are parts in the state space of a cognitive system, and the notion from the theory of artificial neural networks that memories are coded by attractors in the state space of the network^44^. This idea could also reconcile two conflicting views on the number of neurons involved in coding of a single memory^45^, with the “distributed coding” suggesting that many neurons are involved, against the “sparse coding” insisting on the involvement of only a small number of them. Within the same neural circuit, different features of the velocity field co-exist in the same state space formed by *all* neurons of this circuit. If during retrieval of a certain memory most of the neurons are silent, this only means that the given memory is located in some corner of the joint state space of all neurons, so the silence of some neurons is also part of the code.

The mechanism of memory formation in standard models of neural networks of the type (5)-(6) can be interpreted in terms of the velocity field as follows. The equations (5)-(6) jointly represent a standard non-autonomous dynamical system (4). When the stimulus ${\boldsymbol{\eta }}(t)$ vanishes, its velocity field $(\hat{{\boldsymbol{a}}}+{\boldsymbol{\eta }}(t),{\boldsymbol{b}})$ instantly reverts to its core form $(\hat{{\boldsymbol{a}}},{\boldsymbol{b}})$ and keeps no memory trace from the stimulus. But memories about past stimuli are preserved in its part $\hat{{\boldsymbol{a}}}$, at least for a while. This is due to the fact that after ${\boldsymbol{\eta }}(t)$ takes zero value, the connections ${w}_{1},\ldots ,{w}_{M}$ evolve from the values they had immediately before this event slowly, and thus the shape of $\hat{{\boldsymbol{a}}}$ changes very slowly and keeps memory traces for a long time.

#### Self-organizing velocity field

In this Section, we consider the conventional brain models focussing on the properties of the velocity field that steers neural behavior, and hypothesize the self-organising nature of the brain’s velocity field. Based on that, we propose an alternative conceptual model of a cognitive system.

In phenomenological brain models (5)-(6), as well as in their more biologically realistic versions, the intricacy of interaction between the neurodynamics governed by ***a***, the synaptic plasticity governed by ***b***, and the sensory stimuli ${\boldsymbol{\eta }}$, is what makes it so difficult to uncover the fundamental principles behind brain functioning, even when one knows the details of the mechanisms involved. Here we attempt to see through these intricacies and establish that they distill into the spontaneous time-evolution of the brain’s velocity field, which is affected by sensory stimuli. Indeed, the varying connections ***w***(*t*) and stimuli ${\boldsymbol{\eta }}(t)$ jointly ensure that the velocity field ***a***, which controls neural variables ***x*** in (5), de-facto changes with time *t*. This can be formally written as

$${\boldsymbol{a}}({\boldsymbol{x}},{\boldsymbol{w}}(t),{\boldsymbol{\eta }}(t))=\tilde{{\boldsymbol{a}}}({\boldsymbol{x}},t\mathrm{).}$$

More accurate brain models should reflect the plasticity of other parameters in addition to ***w***(*t*), such as variable membrane conductancies^16^. It is useful to shift the focus from the physical structure of the brain to the brain’s velocity field, because the latter is the (mathematical) entity directly controlling the observable neurodynamics, and the structure of this field could explain the occurrence of the given neural firing patterns, provided that the respective brain model is sufficiently realistic.

In neuroscience it is generally assumed that the the plastic parameters of the brain evolve according to some in-built *deterministic* rules, some of which are already established and incorporated into the standard brain models, such as Hebbian learning rule for connections^17^. Moreover, they evolve *spontaneously* in a self-organized manner, in the sense that no external manipulator sets their values or dictates how they change. Certainly, the rules ***b*** in (6) directly link connections ***w*** only with neural activity ***x*** and affect the way the brain’s velocity field $\tilde{{\boldsymbol{a}}}={\boldsymbol{a}}$ evolves only indirectly. However, their assumed determinism and the self-organized nature give us the reason to take a conceptual *leap* and to hypothesize that evolution of the whole of the brain’s velocity field could obey some deterministic rules, too. At this stage, the existence of such rules for the brain is only a conjecture.

From now on, when considering the brain as a dynamical system, we regard its plastic physical architecture as an *instrument* for the creation of its plastic velocity field. The available experience of modelling with dynamical systems teaches us that the rigidity of the physical architecture of the device (e.g. of a neural network) fundamentally restricts the plasticity of the velocity field of its model, even if its parameters (e.g. connections) are allowed to change without restrictions. In our next step, we conceptually *disconnect* the evolving velocity field $\tilde{{\boldsymbol{a}}}$ from the physical mechanisms responsible for its creation and evolution, such as direct perturbation or variability of its control parameters, and focus only on this field and its properties. So we eliminate from consideration any factors that restrict the plasticity of the velocity field, such as the physical architecture or the parameters of the underlying device, and consider the velocity field and its evolution on their own.

As a candidate for a cognitive system, we consider a general dynamical system with an evolving velocity field $\tilde{{\boldsymbol{a}}}$,

7 $$\frac{{\rm{d}}{\bf{x}}}{{\rm{d}}t}=\tilde{{\boldsymbol{a}}}({\boldsymbol{x}},t\mathrm{).}$$

In order to describe a biological system, at any time *t* the function $\tilde{{\boldsymbol{a}}}$ would need to have such a shape, that Equation (7) would represent a nonlinear dissipative dynamical system describing evolution of ***x***. Assume that our hypothesis about the existence of deterministic rules governing evolution of the brain’s velocity field is correct, and recall that the brain is the only device known to perfom real cognition. We link these two considerations and formulate our alternative conceptual model of a cognitive system. Namely, we suggest that in a certain device describable by (7), cognition could amount to the existence of some deterministic rules which appropriately specify how the field changes with time and how it is affected by stimuli.

Velocity fields evolving by themselves according to some pre-defined rules, rather than due to external time-varying perturbations or to time-variation of control parameters, have not been previously considered in the theory of dynamical systems. In order to formulate our conceptual model of cognition mathematically, we need to introduce such fields into consideration. For the purposes of this model, we assume that the field can be as plastic as we like, i.e. it can in principle take any shape and to develop any number of minima and maxima within any given range of ***x*** while staying smooth. We also assume that we can freely choose the rules of its time-evolution to satisfy our needs. We set aside the question how this can be implemented in practice and pose the problem at a conceptual level only. Thus, the spontaneous evolution of the velocity field $\tilde{{\boldsymbol{a}}}$ according to some deterministic rule ***c*** and affected by the stimulus ${\boldsymbol{\eta }}(t)$ can be expressed by the following equation

8 $$\frac{\partial \tilde{{\boldsymbol{a}}}}{\partial t}={\boldsymbol{c}}(\tilde{{\boldsymbol{a}}},{\boldsymbol{\eta }}(t),{\boldsymbol{x}},t\mathrm{).}$$

Equations (7)–(8) epitomize our *alternative conceptual model* of a cognitive system. To understand the meaning of equation (8), one can compare it with a conventional dynamical system (4) affected by stimulus ${\boldsymbol{\eta }}(t)$. Equation (4) states that a vector ***x*** changes with time *t* at a rate and in the direction determined by ***v***, while ***v*** depends on the current value of ***x*** and on ${\boldsymbol{\eta }}$. By analogy, equation (8) declares that the velocity field $\tilde{{\boldsymbol{a}}}$ of (7) changes with time *t* in all locations ***x*** simultaneously, at a rate and in the direction determined by ***c***. At time *t*, these rate and direction depend both on the current shape of $\tilde{{\boldsymbol{a}}}$, and on the stimulus ${\boldsymbol{\eta }}$, might differ at different locations ***x*** in the state space of (7), and might explicitly depend on time *t*. Given that, unlike ***v*** in (4), the field $\tilde{{\boldsymbol{a}}}$ depends both on *t* and on ***x***, in (8) we use its partial derivative with respect to *t* rather than a straight one, so this equation is technically a PDE.

In (8) we treat the velocity field $\tilde{{\boldsymbol{a}}}$ as some vector function of a vector argument ***x***, as well as of *t*. In contrast to the traditional paradigm of dynamical systems, in which the state point in the state space evolves spontaneously according to some *fixed* rules (given by ***v*** in (4)), with (8) we take a conceptual step forward and suggest that the rules $\tilde{{\boldsymbol{a}}}$ of (7) themselves can evolve spontaneously according to some other rules ***c***. This is principally different from a non-autonomous system (4), in which evolution of the velocity field ***v*** is induced merely by external perturbation ${\boldsymbol{\eta }}$ and is not spontaneous. In (8) even without the stimulus, i.e. when ${\boldsymbol{\eta }}={\bf{0}}$, $\tilde{{\boldsymbol{a}}}$ evolves spontaneously according to the rules ***c***. Importantly, we assume that while the stimulus ${\boldsymbol{\eta }}(t)$ in (8) can be random, the rules ***c*** are deterministic. Note, that the system (7)–(8) cannot be obtained with conventional augmentation (or extension) of the state space of a non-autonomous dynamical system, which is often used in control theory, as explained in the Supplementary Note, Section 3.

The model (7)–(8) can be regarded as a special case of the brain model only if our guess about the existence of deterministic rules governing the spontaneous evolution of the brain’s velocity field is proved true. Also, we observe that all neural models share one feature: their state variables are fundamentally bounded. Namely, in physiologically motivated models, voltages and currents can take values only within a range determined by polarization-depolarization processes, while phenomenological models have bounded values of firing frequency^46^. This means that the state space of any model of a neural network is enclosed in a multi-dimensional box. For our conceptual model of a cognitive system we do not need to limit ourselves to the bounded state space, and allow the state variables to vary without bound.

Obviously, not any evolution rules ***c*** can endow the system (7)–(8) with cognitive functions. The mechanism of the field $\tilde{{\boldsymbol{a}}}$ evolution should permit formation of memories as traces left on $\tilde{{\boldsymbol{a}}}$ by the stimulus ${\boldsymbol{\eta }}(t)$, which should not immediately disappear when the stimulus ceases.

### Artificial neural networks: inspirations and limitations

Here, the key principles of memory formation in artificial neural networks are overviewed, which will inspire us to construct a simple cognitive dynamical system of the form (7)–(8). At the same time, our model will avoid the fundamental limitations of neural networks mentioned below.

In the theory of artificial neural networks it has been proposed that memories are represented as attractors arising in the state space of a neural network as a result of choosing appropriate values of connections, see review^44^ and^47^. However, given that neural networks are strongly non-linear systems, it is generally impossible to predict or to control where exactly in their state space the next attractor appears or disappears when connections change their values.

Another well-known limitation of the artificial neural networks is the boundedness of their memory capacity^48^. As a result, memories themselves are short-lived^49,50^, since the formation of a new memory is possible only after the disappearance of an old one. Moreover, neural networks are prone to the uncontrollable formation of spurious attractors not representing any valid memories^51^.

Finally, while the values taken by the stimuli can in principle lie in a very wide range, the states of both the biological and the artificial neural networks are strictly bounded, i.e the available state space lies within a certain box^46^. This issue contributes to the considerable ambiguity in how the input patterns could be represented by the states of the neural network. The traditional approach used in artificial neural networks is to artificially rescale the values of stimuli to the range of values taken by neural variables. However, it seems unlikely that the same approach is utilized in biological neural networks, since it requires an a-priori knowledge of the ranges of values taken by the neural variables and by the sensory signals, while these could in principle vary in time unpredictably.

Artificial neural networks can learn in various styles, but the style which best mimics the performance of the brain is learning without supervision and online^52^, where there is no teacher and the knowledge held by the neural network is continually updated by the incoming flow of data.

### A simple example of a self-organizing velocity field

In this Section we support our alternative conceptual model of a cognitive system (7)–(8) by introducing a simple example of such a system and validating it using simulated and musical stimuli.

In constructing our example, we reverse the traditional approach of the theory of learning in neural networks. Namely, instead of trying to reveal how the given cognitive (usually neuromorphic) system changes its behavior as a result of the change in its parameters while processing stimuli, we try to imagine how an ideal cognitive system *should* adjust its behavior in response to stimuli, so that it does the same job as a typical neural network, but has none of its unavoidable faults. Namely, we formulate a simple *non-neuromorphic* mathematical model of the form (7)–(8), capable of forming memories and retaining them after the stimulus has stopped. Like in the conventional artificial neural networks, our model will represent memories as attractors. However, unlike in the neural networks, these attractors will spontaneously form at the controllable and predictable locations in the state space determined by the properties of the stimuli. Just like artificial neural networks reproducing the learning style of the brain, our model will perform unsupervised learning and will be capable of learning online. With this, unlike in neural networks, there will be no room for spurious memories, and the memory capacity of our model will be in principle unlimited.

To prove the principle, we choose the fist part (7) of our model to be of the simplest form and to represent a gradient dynamical system^53^

9 $$\frac{{\rm{d}}{\boldsymbol{x}}}{{\rm{d}}t}=\tilde{{\boldsymbol{a}}}({\boldsymbol{x}},t)=-\nabla V({\boldsymbol{x}},t),$$

in which the velocity field $\tilde{{\boldsymbol{a}}}$ is the negative of the gradient ∇ of a scalar energy function *V*. Here ***x*** represents in the *N*-dimensional state space the state of the cognitive device, which evolves in time and hence exhibits the observable behavior. Interestingly, the simplest artificial neural networks consisting of identical neurons with symmetric couplings described in^34^ can also be reduced to the form (9), unlike the more realistic models in which couplings are non-symmetric. By analogy with artificial neural networks, ***x*** codes a certain pattern. However, unlike in neural networks, ***x*** is not bounded, and therefore the stimulus values do not need to be rescaled. The state point in (9) behaves like a massless particle placed in a potential energy landscape V(***x***, *t*): from any initial condition it spontaneously moves towards the relevant local minimum, provided that this minimum exists. The attractors in gradient systems can only be of the fixed point type and located at the minima of *V*. We assume that *V* is plastic and undergoes continual reshaping.

**Figure 2:**
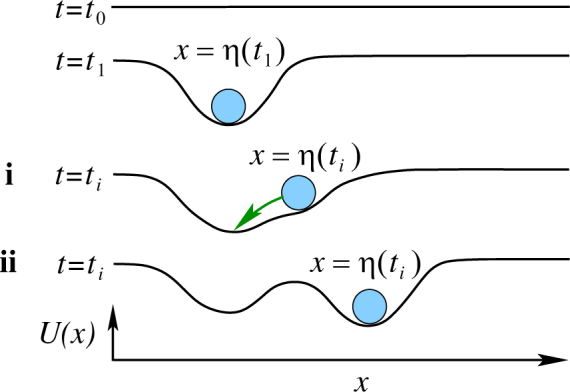
Idea of a plastic energy landscape. Illustration of the idea of the plastic energy landscape by analogy with memory foam. For a one-dimensional “foam” stretched in *x* direction, assume that initially it is flat and described as $U(x,\,\mathrm{0)}=0$ (see *t* = *t*_0_). If the quicksilver drop lands onto the “foam” at position $x=\eta$, the landscape is deformed: a dent appears, which is the deepest exactly at $x=\eta$, and gets shallower at larger distances from $\eta$ (see *t* = *t*_1_). Thus the “foam” will learn about the occurrence of the drop and of its position. For more detail see text.

Below we derive an equation describing the spontaneous evolution of the energy landscape *V* being affected by the random stimulus. To explain this process, it is helpful to employ a loose analogy with the “memory foam” often used in orthopedic mattresses: this foam takes the shape of a body pressed against it, but slowly regains its original shape after the pressure is removed. We convey the idea using the landscape in a one-dimensional space, but it can be extended to the space of any dimension. It helps to use an auxiliary function *U*(*x*, *t*) describing the “foam” profile depending on a single spatial variable *x* and on time *t*, as illustrated by Fig. 2. Assume that the landscape is elastic with elasticity factor *k* that models the capacity of the system to forget. Here, we make a simplified assumption that the deeper the dent at the position *x* is, the faster the landscape tries to come back to *U* = 0. However, the forgetting term can be modelled in a variety of ways, depending on what the situation requires.

Let the stimulus act as a sequence of quicksilver drops, which at successive moments in time ${t}_{1},{t}_{2},\ldots ,{t}_{i},\ldots$ fall on the soft surface at positions $x=\eta ({t}_{i})$. Namely, each drop lands, locally deforms the landscape and slides towards the local minimum while gradually evaporating. Starting from a flat landscape with no features and thus free of memories (Fig. 2, $t={t}_{0}$), the first drop creates a dent centered at $x=\eta ({t}_{1})$ which becomes the first memory (Fig. 2, $t={t}_{1}$). A subsequent drop lands at a different spot $x=\eta ({t}_{i})$ and deforms *U* in one of two ways. (i) If $\eta ({t}_{i})$ is sufficiently close to an earlier stimulus, the drop amends the respective existing dent (Fig. 2, **i** $t={t}_{i}$) and slides to its bottom. Thus $\eta ({t}_{i})$ both amends the existing memory and is recognised as belonging to the same memory, just like perception of different shades of yellow could form a single memory of a yellow color. Alternatively, (ii) if $\eta ({t}_{i})$ is very distinct from any existing memory, it forms its own memory (Fig. 2, **ii** $t={t}_{i}$), and the drop remains at the bottom of the respective dent while evaporating. This process is repeated as more stimuli arrive. The shaping mechanism operating here resembles the kernel density estimation used in statistics^54^, however, here it is performed in a continuous time domain and under more general assumptions.

Consider how *U*(*x*, *t*) changes over a small, but finite time interval Δ*t*:

10 $$U(x,t+{\rm{\Delta }}t)=U(x,t)-g(x-\eta (t)){\rm{\Delta }}t-kU(x,t){\rm{\Delta }}t,$$

where *g*(*z*) is some non-negative bell-shaped function describing the shape of a single dent left by the quicksilver drop, e.g. a Gaussian function

11 $$g(z)=\frac{1}{\sqrt{2\pi {\sigma }_{z}^{2}}}\exp (-\frac{{z}^{2}}{{\sigma }_{z}^{2}})\mathrm{.}$$

In (10) move *U*(*x*, *t*) to the left-hand side, divide both parts by Δ*t*, and take the limit as ${\rm{\Delta }}t\to 0$, to obtain

12 $$\frac{\partial U(x,t)}{\partial t}=-g(x-\eta (t))-kU(x,t\mathrm{).}$$

It can be shown that for some arbitrary $\eta (t)$ the solution *U*(*x*, *t*) has a linear trend and tends to −∞, i.e. it behaves as a linearly decaying function of *t* with superimposed fluctuations. To eliminate this trend for *t* > 0, we make the following change of variables

$$V=\frac{U}{t},\quad \frac{\partial V}{\partial t}=\frac{1}{t}(\frac{\partial U}{\partial t}-V),\quad \frac{\partial U}{\partial t}=t\frac{\partial V}{\partial t}+V,$$

and rewrite (12) as follows

13 $$\frac{\partial V}{\partial t}=-\frac{1}{t}(V+g(x-\eta (t)))-kV\mathrm{.}$$

**Figure 3:**
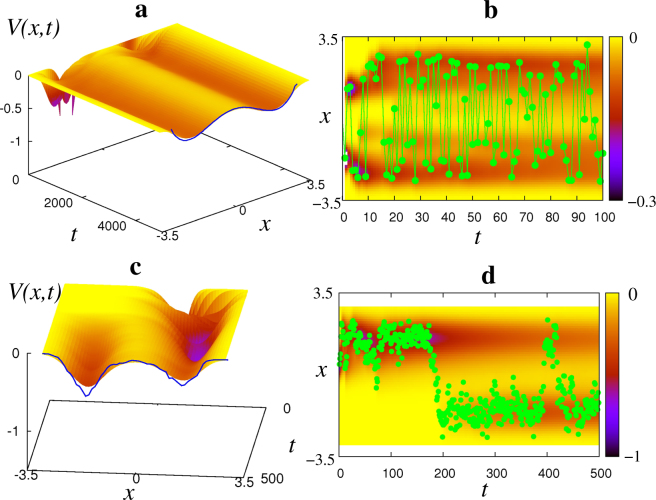
“Learning” energy landscape with simulated stimuli. Evolution of the energy landscape *V*(*x*, *t*) as the random stimulus is applied by numerically simulating equation (13) with *k* = 0: (**a**,**c**) 3D view; (**b**,**d**) projection of *V*(*x*, *t*) onto (*x*, *t*) plane shown by color, and the stimulus applied–by filled circles. In (**a**,**c**) the probability density distribution of stimulus is given by solid line at the front. In (**a**,**b**) the consecutive values of the stimulus are uncorrelated, and in (**c**,**d**)–correlated.

In the *N*-dimensional phase space (13) becomes

14 $$\frac{\partial V}{\partial t}=-\frac{1}{t}(V+g({\boldsymbol{x}}-{\boldsymbol{\eta }}(t)))-kV,$$

where ***x*** and ${\boldsymbol{\eta }}(t)$ are vectors. Within model (13), or the more general model (14), the landscape *V* and the respective velocity field of equation (9) progressively smooth out and, if ${\boldsymbol{\eta }}(t)$ is produced by a stationary process, stabilize. This is illustrated in Fig. 3 for a one-dimensional system subjected to a random stimulus $\eta (t)$ with a two-peak probability density function, usually called “distribution” for brevity. Equations (9), (14) represent the model of a simple DS with plastic self-organizing velocity field.

To model online unsupervised learning typical of the brain, when automatic creation of categories from input data (unsupervised learning) occurs simultaneously with attributing the incoming data to a certain category (pattern recognition), the stimulus ${\boldsymbol{\eta }}$ needs to play two roles. Namely, besides allowing the stimulus ${\boldsymbol{\eta }}(t)$ to deform the landscape in (14) and therefore the velocity field of (9), which model unsupervised learning, we need to reset the state ***x***(*t*) to the current value of ${\boldsymbol{\eta }}(t)$ at regular time intervals to allow the system to recognize the respective pattern by approaching the relevant attractor.

### Testing the model

Here we assess cognitive abilities of the model (9), (14) when it is presented with various random input signals generated numerically. For simplicity, we start from testing the one-dimensional system, whose landscape evolution is described by (13), and will illustrate the process of unsupervised learning as spontaneous shaping of the landscape while processing a stream of input data.

Assume that this system receives time-varying stimulus $\eta (t)$, whose values are randomly taken from two categories of non-equal size. Compare the performance of this system with two different kinds of such input: when consecutive values are completely independent, and when they are correlated. Figure 3 shows evolution of the landscape *V*(*x*, *t*) in each of these cases (see Supplementary Note, Section 4, for the full explanation of how the simulated stimuli were obtained). The values of stimuli arriving at consecutive time moments are depicted by filled circles in **b** and **d**, and their distributions have similar two-peak shapes, whose negatives are shown by the solid lines at the front of **a,c**. The stimulus illustrated in Fig. 3a,b consists of independent values of a random variable with a double-peak distribution (shown in **a** by a solid line), obtained by applying a nonlinear transformation to Gaussian white noise. In the stimulus in **c,d** the consecutive values are correlated and are generated by numerically solving a stochastic differential equation describing the motion of a particle in a non-symmetric double-well potential with large viscosity, to which Gaussian white noise is applied^55^. The negative of the distribution of this stimulus is shown in **c** by solid line and shows two peaks corresponding to two categories of data.

For both of these simulations, in *g*(*z*) described by (11) we used ${\sigma }_{z}=\sqrt{0.1}$, which was small enough to resolve two categories of data. One can see that eventually both landscapes shape into the respective distributions of stimuli (taken with negative signs), but when the stimulus values are uncorrelated, the convergence is faster. If the random process producing the stimulus is not stationary, *V* evolves into a *time-averaged* distribution of the input.

Here, every well of the resultant landscape represents a basin of attraction of a fixed point attractor at its bottom. The gradient of the landscape within every basin of attraction represents a single element of the one-dimensional velocity field in (13) and codes one memory, or category of data. The respective attractor is formed where the most typical (i.e. the most probable) example from each category is located, corresponding to the peak in the distribution. In both cases considered, two categories of data are automatically transformed into two memories coded as different elements of the velocity field within different basins of attraction of non-equal size. Unlike in neural networks, the mechanism (13) (more generally (14)) of memory formation is very transparent and allows one to predict exactly how the existing memory landscape will be adjusted when the next stimulus appears at the given location ***x***.

### Application to musical data

**Figure 4:**
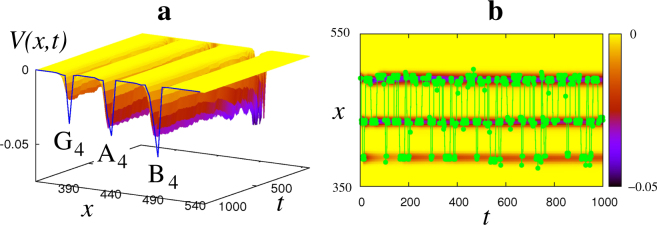
Musical notes recognition. (**a**) Evolution of the landscape *V*(*x*, *t*) in response to a musical signal performed by an amateur musician. Local minima that develop eventually are very close to the frequencies of the musical notes *G*_4_, *A*_4_ and *B*_4_ that enter the song. (**b**) Filled circles show the actual values of the input, and the shade of the background shows the depth of the landscape.

Next, we present our model with a challenge closer to real life. Namely, we test how the system (9), (14) automatically discovers and memorises musical notes and phrases. A children’s song “Mary had a little lamb” was performed with a flute by an amateur musician six times. The song involves three musical notes (*A*, *B* and *G*), consists of 32 beats and was chosen for its simplicity to illustrate the principle. The signal was recorded as a wave-file with sampling rate 8.kHz (see Supplementary Audio A1). In agreement with what is usually done in speech recognition^56^, the short-time Fourier Transform^57^ was applied to the waveform with a sliding window of duration $\tau =0.75$ s, which was roughly equal to the duration of each note. The highest spectral peak was extracted for each window, which corresponded to the main frequency *f* Hz of the given note. A sequence of frequencies *f*(*t*) was used to stimulate the system (13). Note, that each value of *f*(*t*) was slightly different from the exact frequency of the respective note. The reason is the natural variability of the notes introduced by a human musician and by the process of recording the music with the inevitable measurement noise present, so the signal *f*(*t*) was in fact random, as seen from Fig. 4b.

Firstly, we illustrate how individual musical notes can be automatically identified. A one-dimensional system (13) received the signal $\eta (t)=f(t)$, resampled to 8.Hz to save computation time. The function *f*(*t*) can be seen as a realization of a 1st-order stationary and ergodic process *F*(*t*), consisting of infinitely many repetitions of the same song, which we observe during finite time. This process has a one-dimensional distribution ${p}_{1}^{F}(f)$, which does not change in time. A Gaussian kernel *g*(*z*) was used with ${\sigma }_{z}=\sqrt{5}$ Hz. As shown in Fig. 4a, the energy landscape converges to some distribution (taken with negative sign) shown by the solid line. The most probable frequencies are automatically discovered as the energy minima as follows, with figures in brackets showing the exact frequencies of the respective musical notes: 434 Hz (440 Hz) for *A*_4_, 490 Hz (493.88 Hz) for *B*_4_, and 388 Hz (392 Hz) for *G*_4_.

Secondly, we show how the model (14) can discover and memorize the whole musical phrases in the melody, rather than separate notes. To achieve this, we need to present the system with these phrases coded by some vectors ${\boldsymbol{\eta }}$. Let every musical phrase consist of four beats, i.e. of four notes of equal duration $\tau =0.75$ s appearing in a strict order. It is natural to code such a phrase by a vector having these notes as components. Given that the beginnings of consecutive notes are separated by the notes’ duration *τ*, to ensure that at any time *t* the stimulus vector ${\boldsymbol{\eta }}(t)$ contains all four notes, its components should be the four values of *f*(*t*) describing the melody separated by *τ*, i.e. ${\boldsymbol{\eta }}(t)=(f(t),f(t+\tau ),f(t+2\tau ),f(t+3\tau ))$. The procedure of creating a vector with coordinates made of the delayed versions of the same signal, if this signal is generated by some dynamical system, is a standard method of reconstructing phase trajectories from experimental data, and is called “delay embedding”^58^. However, here technically the same procedure is used for a different purpose of coding temporal patterns present in the signal, irrespective of how this signal is produced. To identify the four-beat phrases, a 4D “foam” was used.

To interpret the results of this part, we can regard ${\boldsymbol{\eta }}(t)$ as a realization of a fourth-order stationary and ergodic vector random process ***H***(*t*) (which we observe during finite time) with a four-dimensional distribution ${p}_{4}^{{\boldsymbol{H}}}({f}_{1},{f}_{2},{f}_{3},{f}_{4})$. In simulations of equation (14) we used a multivariate Gaussian kernel *g* with ${\sigma }_{z}=\sqrt{5}$ Hz in all of its four variables.

**Figure 5:**
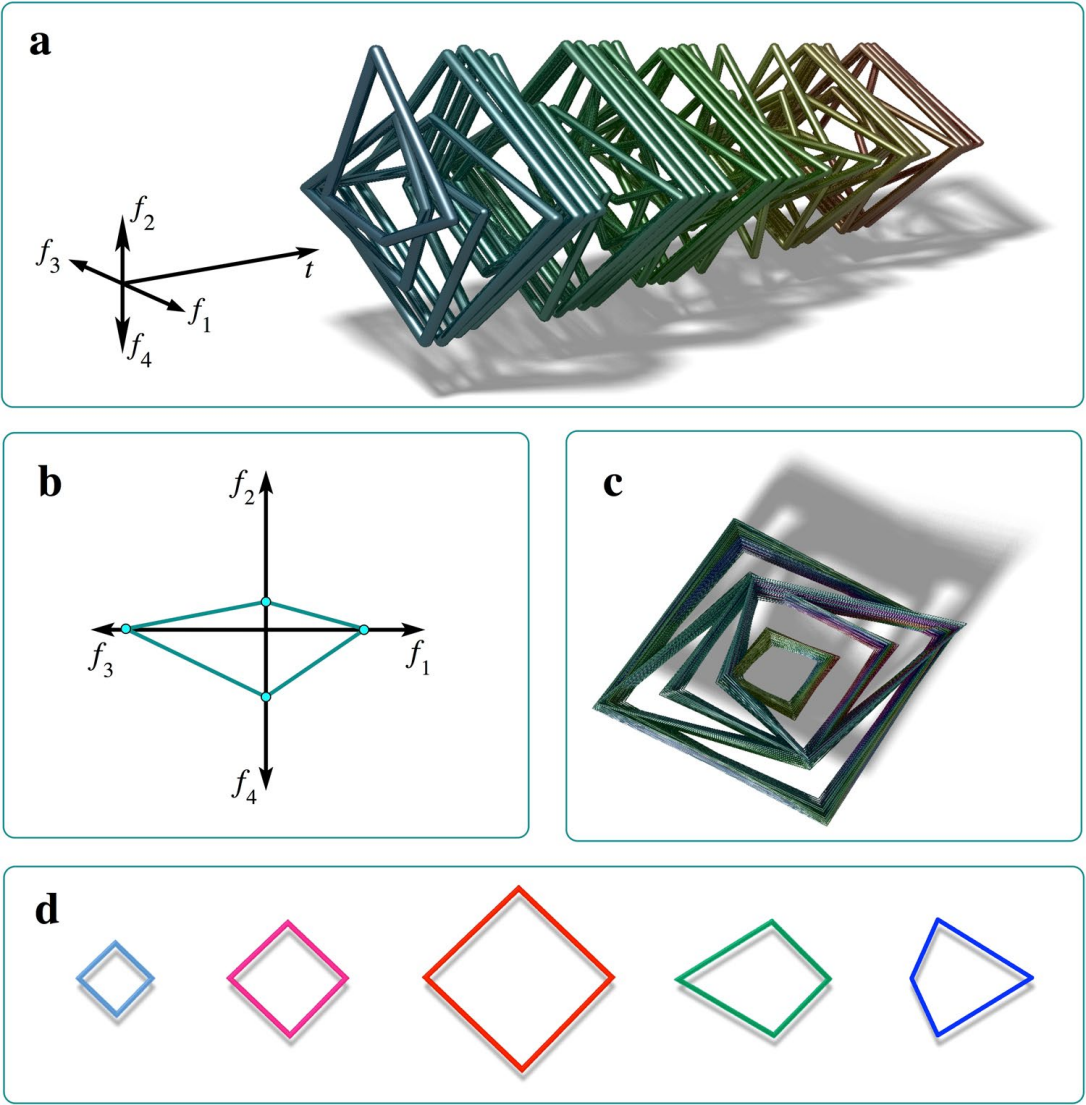
Automatic discovery and memorization of musical phrases. (**a**) The sight of music: a sequence of melody pieces represented as polygons, whose formation is illustrated in (**b**). The shade is only used to enhance visualization and carries no additional information. (**b**) Representing a four-note phrase with a polygon. (**c**) The resultant snapshot of the four-dimensional landscape *V*(***x***, *t*). The function *V* is shown with the smallest values on the top for visualization purposes. The sharp edges on the top represent the most typical musical patterns. (**d**) The most typical musical phrases automatically detected by the system (9), (14).

One cannot visualize evolution of either a four-dimensional time-varying input vector, or of a 4D landscape, in the same way as evolution of a scalar input and of a 1D landscape shown in Figs 3–4. Therefore, we need to find alternative representations for them. Firstly, we need to find a way to visualize a four-dimensional vector. For this purpose, we take four half-axes, attach their origins to the same point, and arrange them with the same angular distance *π*/2 from each other (Fig. 5b). Assuming that all frequencies are positive, i.e. *f*_*i*_ > 0, for any feasible input vector ${\boldsymbol{\eta }}=({f}_{1},{f}_{2},{f}_{3},{f}_{4})$ we put four points with coordinates *f*_*i*_ on each of the half-axes, and connect them by straight lines. This way, any feasible 4-beat musical pattern can be represented by a planar polygon, in this case by a tetragon. The same method can be used to represent on a plane a vector of any dimension, provided that its components are positive.

The sequence of four-beat musical phrases forming the melody can be visualized as a sequence of polygons placed at the time moments when the first note of each phrase sounds, as shown in Fig. 5a. Now, to visualize a scalar function *V* of the vector ${\boldsymbol{\eta }}$ at a single time moment, the polygon representing the given ${\boldsymbol{\eta }}$ can be placed at an altitude equal to the value of $V({\boldsymbol{\eta }})$. In Fig. 5c a snapshot of *V* is visualized after the equation (14) finished processing its four-dimensional musical input, with the deepest polygons shown on top for better clarity. Because polygons projected on the plane of the figure overlap, it might be somewhat difficult to visually identify the highest ones, which code the most probable musical phrases. However, these can be found by numerical simulation of equation (9) by trying randomly chosen initial conditions, from which the phase trajectories converge to the local minima. Five polygons impersonating the most probable musical phrases are given in smaller scale in Fig. 5d. The performance of equation (14) is also illustrated with the Supplementary Audio A2, which contains the whole song reconstructed from the four-beat musical phrases, which the model recognized and memorized automatically. Comparison of the melody in A2 with the original one in A1 reveals that our model discovers and memorizes the musical phrases correctly.

### Data availability

The datasets generated during and/or analysed during the current study are available from the corresponding author on reasonable request.

## Discussion

To summarize the findings of this paper, by analysing conventional dynamical models of the brain in the form of neural networks, we shift the focus from their behavior to the *cause* of their behavior. In a dynamical system, the behavior is caused by its velocity vector field, which explicitly instructs the system what to do in every feasible situation. In a realistic dynamical model of a physical device, the velocity field is essentially the physical architecture of the device expressed in mathematical language (see Supplementary Note, page 3, paragraphs 2–3). We figure out that the physical plasticity of the brain gives rise to the plasticity of its velocity field. Given the uniqueness of the brain both as being a truly cognitive device, and as having a plastic velocity field, we link these two features and suggest that the former might be enabled by the latter. We also suggest that the rules governing evolution of the brain parameters (e.g. connections and excitability) serve as an instrument to enable the brain’s velocity field to evolve in a manner needed for cognition. Moreover, assuming the existence of determinstic laws governing the *physical* plasticity of the brain, we take a conceptual leap and hypothesize that there could also exist deterministic laws governing the spontaneous evolution of the brain’s velocity field. Regardless of the validity of this guess, on its basis we propose an alternative conceptual model of a cognitive system. This model states that, in mathematical terms, cognition could amount to the spontaneous self-organized evolution of the velocity field of a cognitive device according to some in-built appropriate rules, which is affected by sensory stimuli. Dynamical systems with such velocity fields have been unknown to date, so we introduce them here. We construct a very simple example of a non-neuromorphic system of this novel type and demonstrate its basic cognitive abilities, thus supporting our mathematical hypothesis about the possible nature of cognition.

On the way to formulating the conceptual model of cognition, we propose our version of a solution to the problem of memory representation in the brain: we suggest that memories are embodied in the features of the brain’s velocity field. The advantage of this idea is that, rather than rejecting any previous memory paradigms, it unifies them, showing that they represent different manifestations of the same velocity field.

When constructing our sample cognitive model, we tried to overcome the traditional issue of artificial neural networks being the impossibility to predict where in the state space the new memory will appear, and how exactly the existing memories will be changed, when the new stimuli arrive. The mechanism of memory formation in our sample model is transparent in this sense: individual inputs are automatically and without supervision memorized as imprints on its velocity field at predictable locations. With this, traces from several stimuli belonging to the same category automatically merge together and develop into one large memory imprint. Our system learns online by continually updating its knowledge of the available categories, and is lacking traditional limitations of neural networks, such as spurious or bounded memories. It also reproduces an important feature of human memory: every time a certain memory is recalled from the cue, it is slightly modified^59,60^. This example model could serve a useful metaphor to describe the principles of elementary cognition.

In a more general perspective, the significance of our conceptual model is four-fold. In physico-chemical systems, self-organization has long been associated with spontaneous behavior according to some fixed rules specified by the velocity field, as in e.g. Belousov-Zhabotinsky chemical reaction^61^. Here, we propose to extend the concept of self-organization to the rules of behavior themselves, which constitutes a conceptual advancement within the systems theory. Secondly, spontaneously evolving velocity fields could represent the missing element of the dynamical systems theory directly relevant to cognitive processing, whose absence has been preventing this theory from forming the foundation of cognitive science^14^. Thirdly, our model could prompt the development of artificial cognitive devices based on the proposed principles, which would be different from neural networks or computers. Specifically, it could play the same role as a Turing machine did for a computer. Namely, a Turing machine is a physically impossible device (due to the requirement of an infinitely long tape), and all digital computers ever made have been very different from it in their architecture, but all of them have been implementing the principle of this machine. Similarly, our conceptual model could inspire creation of devices with plastic velocity fields evolving according to the prescribed laws, and some of these laws might lead to cognition. Also, a Turing machine can be viewed as the result of conversion of vague ideas about the nature of human thinking into a mathematically explicit idea that thinking is the process of going from one statement to another according to some pre-defined rules. Despite an enormous success of computers, it is now well appreciated that their working principles are not equivalent to the principles of brain functioning^7^. Our alternative conceptual model of cognition is another attempt to express cognition mathematically, but in different terms. However, in the context of “understanding the brain” our model has some advantage over the Turing machine because, unlike the latter, it can be explicitly linked to measureable brain parameters and variables, and to the observable behavior of the human. Namely, its central object, the velocity field of the brain, is both determined by the brain parameters, and determines the behavior of the neural variables, which in their turn control the behavior of the body.

Fourthly, putting the focus on the brain’s velocity field could help answer the whole set of intertwined open questions about the brain. One of them is the major outstanding problem of neuroscience, namely, the problem of finding an explicit link between the two levels of brain activity: the lower level of cellular processes, and the higher level of cognitive functions and behavior^10,19,62^. There is currently a conceptual gap between these levels, and it is not clear how the latter arises from the former^4,5,63^. To develop the general theory of the brain, one needs to find an appropriate level of its description, which would be intermediate between the two levels mentioned above^1,11^. We argue that this intermediate level, and simultaneously the link between the brain architecture and the behavior, could be the velocity field of the brain.

Indeed, the observable behavior of humans, including bodily movements and verbal utterances, is coordinated by neural firings through activation of relevant muscles^64^. Assume that the brain be described as a dynamical system. Then on the one hand, the velocity field of the brain would be a mathematical incarnation of the force that controls firings of every neuron. On the other hand, the shape of the velocity field of the given brain would be determined by the full set of this brain’s physical parameters. Therefore, the velocity field of the brain would represent a *mediator* between the behavior and the brain architecture. Unlike the behavior or the neuron firings, this mediator field is not observable in full. Namely, an almost direct registration of the velocity field could be possible only along the phase trajectories, which could be reconstructed from the high-quality data of activity of every neuron in the brain using approaches overviewed in^39^ and in Supplementary Note, Section 2. However, this method would deliver only tiny segments of the velocity field, and therefore would be insufficient. Thus, the velocity field cannot be measured in an experiment in full and represents a fundamentally hidden entity. However, it can in principle be reconstructed with some degree of accuracy in the process of building a detailed model of the given brain, while incorporating into this model the data about all the brain parameters, such as connections, membrane conductances of individual neurons, etc. Because these parameters change in time, at different time moments the velocity field of the given brain is different.

Another related question is how to interpret detailed brain data^62,65^, whose collection is in the focus of the ongoing brain research^3,10^ and which consist of (generally plastic) parameters and of variables. Our conceptual model of the brain could offer an answer to this, as well. Namely, as mentioned above, the parameters of the brain, together with its architecture, determine the shape of its velocity field and hence can be used to reconstruct an approximation of this field at every time moment through modelling from the first principles. At the same time, neural activity data are guided by this field and therefore carry some information about its shape.

It has been well appreciated that cognition is an emergent property of the brain^4,18–20^, and at the same time it is a property directly affecting behavior^64^. The velocity field of the brain can be characterized in exactly the same words, but in addition it represents an entity, which can be principally accessed through revealing the brain’s physical architecture and experimentally measuring its parameters. Our proposal to describe cognition in terms of the velocity field of the brain could help expose cognition to direct studies with the tools of physics, chemistry and mathematics.

One other big goal of neuroscience is to understand “the laws, not the details of brain dynamics”^21^ and to build falsifiable models of the brain^6^. Our hypothesis, that cognition could be explained as evolution of the brain’s velocity field according to some laws, as expressed by equations (7)–(8), is currently only a guess, albeit indirectly supported by the performance of a sample cognitive model obeying the same principle. However, (7)–(8) represent a conceptual model of the brain, which could be falsified or verified with the help of detailed brain measurements. Moreover, in case this model is verified, it could be possible to reveal the very laws behind the evolution of the brain’s velocity field.

To do this, first of all, one would need to obtain snapshots of the velocity field $\tilde{{\boldsymbol{a}}}$ of the brain at every moment of time *t* within some sufficiently long time interval. As mentioned above, approximations of this field could potentially be recovered from building the brain model and substituting into this model the values of brain parameters experimentally registered as functions of time. After that, to assess the presence of a deterministic rule ***c*** behind the time-variation of $\tilde{{\boldsymbol{a}}}$, one might be able to adapt and extend the approaches of global reconstruction of dynamical systems from experimental data^39^. Specifically, standard global reconstruction techniques are applicable when one registers the behavior of the dynamical system as either a scalar state variable, or the full state vector, as a function of time (see Supplementary Note, page 8, paragraph 1 for references). Then one can approximately reconstruct the velocity field of the underlying dynamical system, provided the data are of sufficient length and quality. In the task of verifying the validity of (7)–(8), the observation “variable” would be the whole of the velocity field of the brain as a function of time, and one would be looking for the laws behind its evolution, which is a considerably greater challenge. But even before undertaking to solve this problem, one would need to develop technologies to make it possible to record data from the living brain in real time, non-invasively, and with sufficiently high detail and accuracy. In view of the ongoing considerable effort in this direction, it is important to formulate specific questions to ask of the brain data being collected^3,10^, and to use them to test specific hypotheses about the brain function^6,11,62^. The validity of (7)–(8) as a brain model could serve as a candidate question potentially answerable with the help of detailed brain measurements.

If the existence of such laws is confirmed and the laws themselves are discovered, they might represent a part of those sought-after laws of brain dynamics. Also, they could be connected to the mechanisms underlying plasticity of the physical brain parameters, which would deliver the link between the cognitive processes and cellular-level processes.

## Electronic supplementary material

Supplementary Information

Audio file A1

Audio file A2
