# Supplementary material for: Dynamical system with plastic self-organized velocity field as an alternative conceptual model of a cognitive system
Source: Sci Rep. 2017 Dec 5;7:17007. doi: 10.1038/s41598-017-16994-y (PMC5717027; doi:10.1038/s41598-017-16994-y)
Supplement: Supplementary file 1 — Supplementary Information [file 41598_2017_16994_MOESM1_ESM.pdf]

Dynamical system with plastic self-organized velocity field as an alternative conceptual model of a cognitive system

Supplementary Note

Natalia B. Janson[1,*] **and Christopher J. Marsden**[1]

[1] Department of Mathematical Sciences, Loughborough University, Loughborough LE11 3TU, UK

[*] N.B.Janson@lboro.ac.uk

Table of Contents

1. Velocity Vector Field as an Emergent Property of a Device 1
1.1. Construction of a dynamical model: mechanical example 2
1.2. Emergent velocity field of a neural network 3
2. Reconstructing Velocity Field from Experimental Data 5
2.1. Case 1: all state variables are recorded 5
2.2. Case 2: a single realization is recorded 6
3. Comparison of our Conceptual Model with State Space Augmentation 8
4. Simulated Data 10
References 10

1. Velocity Vector Field as an Emergent Property of a Device

In the main text of the paper we mention the principal possibility to obtain an approximation of the velocity field of a spontaneously evolving device, including the one of the brain, if one knows the architecture and the parameters of the device. This can be done by constructing from the first principles a dynamical model in the form (1)/(2), which would deliver the velocity field $\boldsymbol{s}$ automatically. Although the relevant methodology is standard and well established, for the convenience of the reader, in Section 1.1. of this Note we give a brief overview of this approach and demonstrate how it is applied to a simple mechanical system.

Also, in the main text of the paper we point out that the velocity field of a multi-component spontaneously evolving device can be regarded as its emergent property. The concept of emergence dates back to ancient times, and its definition is often summarized in a saying, which paraphrases Aristotle, "The whole is other than the sum of its parts". There exist several more specific definitions of emergent properties, for example, "Many groups or aggregates have properties that are not properties of the individuals of which they are a collection. Such properties are called emergent properties... Emergence: the property of the whole is produced by properties of the parts but is not qualitatively similar."[S1]. An excellent exposition of the concept of emergence can be found in[S2]. Importantly, emergent phenomena arise from *interactions* between the components of a system and cannot be deduced solely from the properties of these individual components, or reduced to their sum[S3]. An example of an emergent property from biology is the ability of the heart to pump blood, whereas individual cells forming the heart are unable to do so. Cognitive functions[4,18–20] and consciousness[S2] are widely appreciated to be the emergent properties of the brain. In the device considered in Section 1.1., without interaction between its parts there is no spontaneous evolution and therefore no velocity field. We show how the velocity field emerges only after the components of this device are put together and start interacting. In Section 1.2. we illustrate a slightly different situation, when every component of the system can evolve spontaneously even without interacting with other components, i.e. possesses an individual velocity field. We show how, after the components are coupled, all individual velocity fields disappear and the global velocity field arises, which is principally irreducible to a sum of the individual velocity fields of the components and hence represents an emergent property of the system.

1.1. Construction of a dynamical model: mechanical example

All devices obey the standard laws of physics, such as those of mechanics and electricity, which come in the form of algebraic or differential equations. When constructing a dynamical model for a man-made spontaneously evolving device with a known architecture, one applies to its various parts the relevant physics laws, and then combines them. In doing so, one needs to choose an appropriate level of the description of the given device and to decide what factors are essential and what factors could be neglected. If the differential equations used are of the "ordinary" type, one can rearrange their terms and, by using suitable variable substitutions, obtain a set of first-order ordinary differential equations of the form (1)/(2) (or (4) for devices experiencing external perturbation) and thus single out the velocity field.

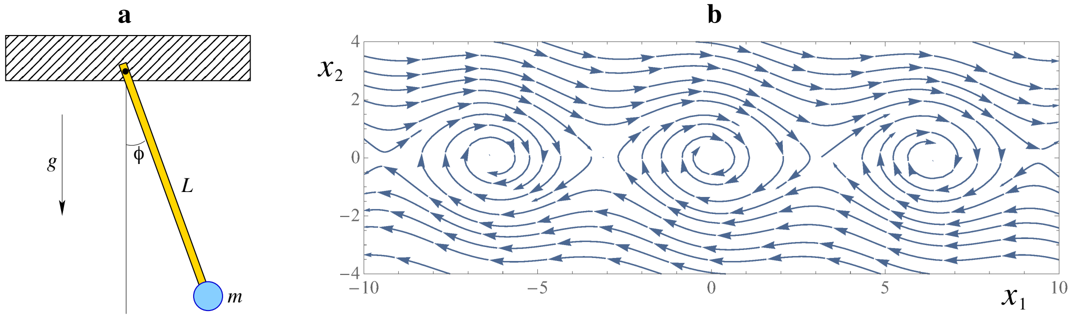

Figure S1. **a** Bob pendulum. L is the length of the rope, m is the mass of the bob, and g is acceleration due to gravity. **b** Velocity field (S1) of the bob pendulum with friction with g/L=1 and λ=0.15.

Below we illustrate the process of construcing from the first principles a dynamical model (1) for one of the best known and the simplest spontaneously evolving mechanical man-made devices, a bob pendulum (Figure S1**a**). Spontaneous swinging of the bob arises only if it is attached to the rope of length L, which in its turn is attached to the support, and if the gravity force with acceleration g is present. If the parts of the pendulum are disconnected from each other, no motion, i.e. no evolution, takes place. Individual parts of the pendulum cannot give rise to, or explain, oscillations, which occur only when all elements are connected and interact. When choosing the appropriate level of detail in modelling such a pendulum, one usually discards thermal fluctuations of individual molecules forming its parts, or the fact that the rope stretches and contracts during oscillations thus changing its length L, and can possibly bend, or that acceleration due to gravity g, strictly speaking, changes with the instantaneous altitude of the bob, or that the bob has a finite volume and mass and is affected by interaction with the surrounding air. Also, the real bob does not necessarily oscillate in a perfect plane. However, for the model one assumes that the pendulum swings in a perfect plane, that the bob has zero size (i.e. represents a point mass) and therefore all effects from molecular fluctuations or interactions with the air are very small and can be neglected, that the rope is massless, and the variable of interest is a macroscopic quantity, the angle ϕ between the rope and the vertical line, which can be registered experimentally. With this, both L and g can be regarded as approximately constant in the given problem. In all real devices, whose size is much larger than the one of typical molecules and much smaller than the one of the bodies travelling in the open space, there will be dissipation of energy through heat. In mechanical systems energy dissipation occurs from various sorts of friction between their parts. In the given pendulum, dissipation occurs from the friction at the pivot and from the air resistance.

We can apply to the bob a famous law of physics, the Newton's second law of motion in the rotational form, which states that the torque (the moment of force) T is equal to the moment of inertia I multiplied by the angular acceleration $\frac{\mathrm{d}^2\phi}{\mathrm{d}t^2}$, i.e. can be described by a second-order ordinary differential equation for ϕ[S4]

$$T = I\frac{\mathrm{d}^2\phi}{\mathrm{d}t^2}.$$

Here, the torque and the moment of inertia can be found using the following algebraic equations, namely, T=$-mgL\sin\phi$ and I=mL^2, respectively. Next, we substitute these algebraic equations into the differential equation above. We also include a term modelling friction force, which is usually proportional to the mass m and to the instantaneous angular velocity $\frac{\mathrm{d}\phi}{\mathrm{d}t}$ of the pendulum with proportionallity factor λ>0. We thus obtain

$$-mgL\sin\phi - \lambda m\frac{\mathrm{d}\phi}{\mathrm{d}t} = mL^2\frac{\mathrm{d}^2\phi}{\mathrm{d}t^2},$$

which after the rearrangement of terms becomes

$$\frac{\mathrm{d}^2\phi}{\mathrm{d}t^2} = -\lambda\frac{\mathrm{d}\phi}{\mathrm{d}t} - \frac{g}{L}\sin\phi.$$

Finally, we introduce the following change of variables, $x_1 = \phi$ and $x_2 = \frac{d\phi}{dt}$, and rewrite the equation above in the form (1)

$$\dot{x}_1 = x_2, \qquad \dot{x}_2 = -\frac{g}{L}\sin x_1 - \lambda x_2,$$

where dot denotes the time derivative, thus singling out the velocity vector field $\boldsymbol{s}$ as follows

$$\boldsymbol{s}(\boldsymbol{x}) = \left(x_2, \left(-\frac{g}{L}\sin x_1 - \lambda x_2\right)\right). \tag{S1}$$

The velocity field $\boldsymbol{s}$ given by (S1) arises from interactions between the parts of the pendulum and is illustrated in Figure S1**b** with g/L=1 and λ=0.15. Note, that without interaction between the pendulum components there is no spontaneous evolution and no velocity field. Thus, the velocity field governing evolution of the pendulum is the pendulum's emergent property.

If one needs to build a model of a spontaneously evolving *electronic* device, one can apply to its various parts the appropriate laws of electric circuits (Kirchhoff's laws)[S5], which come in the form of algebraic equations for currents and voltages. Assuming that these currents and voltages evolve in time, with the help of the well-established relationships, one can express some currents through the time derivatives of voltages and some voltages through the time derivatives of currents, and substitute everything into Kirchhoff's equations. This way, by rearranging terms and by changing variables, one can obtain a dynamical system (1)/(2) describing the device. A famous example of a dynamical model of an electric circuit is a van der Pol system, which describes a generator of self-sustained periodic oscillations[S6]. The same principle can be applied to chemical reactions occurring either in man-made, or in natural and biological systems. Under certain conditions, these can be described using the law of mass action, whose formulation and numerous applications are overviewed in[S7]. Being translated into the language of mathematics, this law takes the form of a system of first-order ordinary differential equations, each expressing the rate, at which concentration of a certain chemical component participating in the reaction changes in time, as a function of concentrations of all components. A remarkable discovery of spontaneous non-damped oscillations of chemical concentrations in homogeneous chemical systems in the course of what we now know as Belousov-Zhabotinsky reactions was followed by the derivation of their dynamical models in the form (1)/(2) (see[S8] for an overview).

When constructing from the first principles (i.e. not empirically) the models of biological systems, the first step is to identify physical and/or chemical processes involved and to apply to their description suitable physics and/or chemistry laws. Often this involves producing an equivalent electric circuit for the whole or for a part of the biological system. After that one can proceed by analogy with non-biological devices and arrive at the dynamical model (1)/(2). A famous example illustrating this approach is a set of Hodgkin-Huxley equations describing firings of a single neuron[S9]. In all cases, the velocity field of the resultant dynamical model ultimately represents a combination of physics and/or chemistry laws acting on various parts of the system, which are assembled into a single structure. Since physics and chemistry laws are the same for all the objects in the Universe, different models will differ only in the way these laws are combined, which is dictated by the architectures of the respective devices. Thus, one can say that the velocity field is a mathematical representation of the architecture of the underlying device, and simultaneously its emergent property.

1.2. Emergent velocity field of a neural network

Here we analyse the situation where every part of a larger system represents a spontaneously evolving device, and thus possesses the velocity field of its own if considered in isolation. However, interaction between these parts erases all individual velocity fields and produces a new global velocity field, which cannot be reduced to, or fully explained through, the velocity fields of the disconnected system components. The system we consider is a neural network.

When modelling a single neuron, one typically reproduces its natural ability to evolve spontaneously, i.e. to fire, and formulates a dynamical system of the form (1) to describe its behavior, such as the one obtained in[S9]. Therefore, one can say that a single neuron possesses its own velocity field, whose approximations with various degrees of accuracy are used in its dynamical models. Note that in biologically realistic neuron models, their dimension is usually quite high (N=4 in[S9]) and the functions $\boldsymbol{s}$ are nonlinear and quite complicated. It is usually a challenge to visualize the velocity field of even a single model of this type, let alone of their network. Since it is critical for us to focus on the velocity field of a neural network, we consider a network consisting of the simplest phenomenological (empirical) neuron models used in[S10], namely

$$\frac{dx}{dt} = -\frac{x}{\mu}.$$

Here, one neuron is represented by a single scalar state variable x, which can be interpreted as the (shifted and scaled) neuron's firing frequency, and μ>0 is a constant parameter characterising a combination of some physical properties of this neuron. The given model can demonstrate only one kind of behavior, namely, from any initial conditions it tends to the only attractor of the system being the stable fixed point at x=0.

If we consider M such neurons, but *do not* couple them, the respective dynamical system reads

$$\frac{\mathrm{d}x_i}{\mathrm{d}t} = -\frac{x_i}{\mu_i}, \quad i = 1, 2, \dots, M,$$

and possess the velocity field

$$\boldsymbol{s}(\boldsymbol{x}) = \left(-\frac{x_1}{\mu_1}, -\frac{x_2}{\mu_2}, \dots, -\frac{x_M}{\mu_M}\right). \tag{S2}$$

This global velocity field is a simple combination of the unchanged individual velocity fiels of the constituent neurons, and does not alter the behavior of any neurons. The velocity field for the two uncoupled neurons is illustrated in Figure S2**a** for μ_1=1, μ_2=1.2.

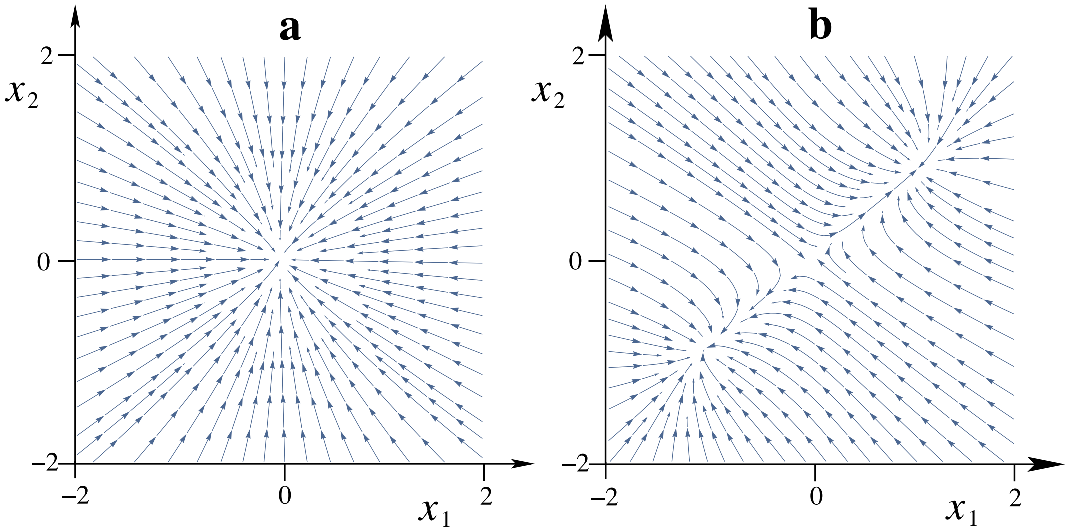

Figure S2. Velocity field of the system of two neurons described by Equation (S3) with N=2. Parameters are chosen as μ_1=1, μ_2=1.2, λ_1=λ_2=1.4, and η_1=η_2=1.4. **a** Uncoupled neurons, i.e. T_{12}=T_{21}=0. **b** Coupled neurons with T_{12}=1.5 and T_{21}=1.

However, if we *couple* M neurons of the above type in a network, its resultant model reads[S10]

$$\frac{\mathrm{d}x_i}{\mathrm{d}t} = -\frac{x_i}{\mu_i} + \sum_{j=1}^{M} T_{ij} g_j(x_j) + \eta_i, \quad g_i(z) = \frac{2}{\pi}\tan^{-1}\left(\frac{\pi\lambda_i z}{2}\right), \quad i = 1, 2, \dots, M. \tag{S3}$$

Here, T_{ij} is the strength of connection between ith and jth neurons, $g_i(z)$ is a function of a sigmoid shape shown in Figure S3, which can be different in different neurons, as described by parameters λ_i. Signal η_i is the input to the ith neuron. In our illustration, following the example of[S10], we set T_{ii}=0 thus eliminating couplings from the neurons to themselves, and η_i=0. The global velocity field $\boldsymbol{s}(\boldsymbol{x})$ of this network is

$$\boldsymbol{s}(\boldsymbol{x}) = \left(\left(-\frac{x_1}{\mu_1} + \sum_{j=1}^{M} T_{1j} g_j(x_j)\right), \left(-\frac{x_2}{\mu_2} + \sum_{j=1}^{M} T_{2j} g_j(x_j)\right), \dots, \left(-\frac{x_M}{\mu_N} + \sum_{j=1}^{M} T_{Mj} g_j(x_j)\right)\right).$$

One can see that it is very different from the velocity field of uncoupled neurons (S2) and represents an emergent property of the network arising from couplings T_{ij}. This emergent velocity field alters the behavior of all neurons in the network drastically and gives rise to multiple new attractors in the global state space of the neural network. An illustration of this phenomenon is given in Figure S2**b** for a network of only two neurons (M = 2), in which the velocity field arising from couplings is compared with the one of a uncoupled system in **a**. Namely, in contrast to **a**, in **b** the attractor at $\boldsymbol{x}$=0 disappeared and two new attractors appeared instead.

The same phenomenon occurs if one couples several neuron models, which are biologically realistic. To summarize, if the brain represents a neural network, which can be modelled as a dynamical system, then the brain's velocity field is an emergent property of the brain arising from interactions between the neurons. In addition, if the model of the brain is accurate,

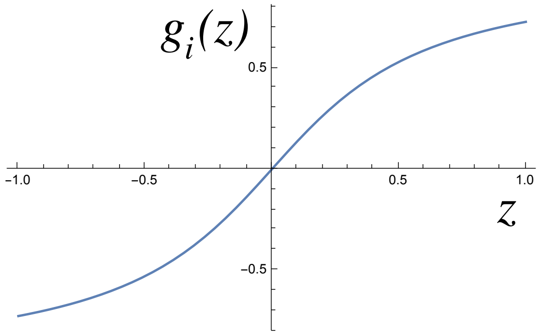

Figure S3. Sigmoid function $g_i(z)$ from Equation (S3) with λ_i=1.4.

this velocity field dictates all the neurons in the brain when to fire. Since the bodily movements and verbal utterances occur thanks to muscular contractions, which are coordinated by motor-neurons, which in their turn are excited by the neurons in the brain, the velocity field of the brain ultimately controls the behavior of the whole body. Being an emergent property of the brain and directly affecting the behavior is what the brain's velocity field has in common with cognition. With this, unlike the cognitive functions, the velocity field is an unambiguously defined object, which can in principle be estimated from experimental measurements of the brain parameters through the process of constructing the model of the brain. These reasons provide an argument towards associating cognition with the velocity field of the brain in order to expose cognition to direct studies with methods of physics and mathematics.

2. Reconstructing Velocity Field from Experimental Data

In the text of the paper we mention the principal possibility of recovering *tiny parts* of the velocity field of the brain directly from the neural activity data. In this Section, for the convenience of the reader, we briefly overview the standard methodology behind reconstruction from the experimental data of the velocity vectors along the phase trajectories. A more comprehensive description of this approach is given in [39]. We consider two cases, namely, when all state variables can be registereed experimentally (Section 2.1.) and when one registers only a single realization reflecting the behavior of the system under study (Section 2.2.).

2.1. Case 1: all state variables are recorded

Suppose we observe the behavior of a spontaneously evolving device, which can be modelled as a dynamical system. Assume that we somehow know the total number N of the scalar state variables of this device, and that we are able to observe all of these variables in an experiment as functions of time t, i.e. we can observe $y_1(t)$, $y_2(t)$, …, $y_N(t)$. We further assume that the dynamical system describing the behavior of our device has the form (1) but is unknown to us. In that case the vector $\boldsymbol{y}(t)=(y_1(t), y_2(t), \ldots, y_N(t))$ represents some particular solution of (1) and therefore satisfies

$$\frac{\mathrm{d}y_1(t)}{\mathrm{d}t} = s_1(y_1(t), \ldots, y_N(t)), \quad \ldots, \quad \frac{\mathrm{d}y_N(t)}{\mathrm{d}t} = s_N(y_1(t), \ldots, y_N(t)). \tag{S4}$$

In all recordings of experimental data the variables being observed are discretized in time with some sampling step $\Delta t=1/f_s$, where f_s is the sampling frequency. The value of f_s needs to comply with the Nyquist-Shannon theorem described e.g. in [40]. Namely, assuming that the signals being recorded are bandwidth-limited, i.e. that their Amplitude Fourier spectra are equal to zero at all frequencies larger than a certain frequency f_b, f_s has to satisfy $f_s \geq 2f_b$. Thus, the experimental data represent a collection of vectors belonging to the phase trajectory $\boldsymbol{y}(t)$, which are available at discrete times $t=k\Delta t$, $k = 1, 2, \ldots, L$, i.e.

$$\boldsymbol{y}(k\Delta t) = (y_1(k\Delta t), y_2(k\Delta t), \ldots, y_N(k\Delta t)), \quad k = 1, 2, \ldots, L, \tag{S5}$$

where L is the total number of the state vectors recorded. Sampled observations of a certain quantity are also called time series.

Although we do not know the velocity field $\boldsymbol{s}(\boldsymbol{x})$ of the model (1) at every point $\boldsymbol{x}$ of the state space, we can reconstruct the velocity vectors $\boldsymbol{s}(\boldsymbol{y}(k\Delta t))$ *along the recorded phase trajectory* by numerically estimating the time derivatives $\dot{y}_i(k\Delta t)=s_i(\boldsymbol{y}(k\Delta t))$ of all state variables at every time moment $k\Delta t$ and forming a vector from them. There exist several methods to estimate the time derivatives from data, and the simplest (and least accurate) method is illustrated in Figure S4. Namely, to estimate the derivative at time $t=t^*$ corresponding to a data point marked by red circle, one can estimate the slope

of a line (cyan line) connecting this point with the preceding one (cyan circle). Thus, the time derivative of the continuous function $y_i(t)$ (magenta) at time $k\Delta t$ can be approximated as

$$s_i(\mathbf{y}(k\Delta t)) = \frac{\mathrm{d}y_i(k\Delta t)}{\mathrm{d}t} \approx \frac{y_i(k\Delta t) - y_i((k-1)\Delta t)}{\Delta t}, \quad k = 2, 3, \ldots, L \tag{S6}$$

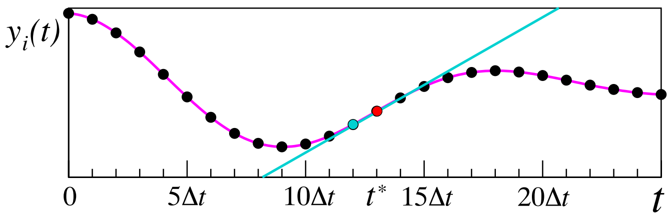

Figure S4. Numerical estimation of a time derivative from a discretized experimental signal $y_i(k\Delta t)$. The continuous function of time $y_i(t)$ is shown by magenta, the data points registered experimentally are shown by black circles, the reference data point at time t=t^* is shown by red circle, and the straight line (cyan line), whose slope is an approximation of the time derivative of $y_i(t^*)$, connects the reference point with the immediately preceding one.

Figure S5 Illustrates reconstruction of the velocity field along the recorded phase trajectory of Rössler system[S11]

$$\dot{x}_1 = -x_2 - x_3, \quad \dot{x}_2 = x_1 + r x_2, \quad \dot{x}_3 = b + x_3(x_1 - c), \tag{S7}$$

where r, b and c are parameters. "Experimental data", also called "realizations", are obtained by the numerical simulation of (S7) with $r = 0.2$, $b = 0.2$ and $c = 6.5$ from initial conditions $x_1(0) = 8$, $x_2(0) = -17$, $x_3(0) = 4.0$, and after discarding transients. The system was integrated with method of Runge-Kutta of the fourth order with intergration step 0.0005, but the solution was resampled for illustration purposes with the sampling step Δt=0.25. In Figure S5**a** three simultaneously recorded realizations are shown during 15 time units. In Figure S5**b** the respective discretized phase trajectory of (S7) is shown in the three-dimensional state space. In order to obtain this trajectory, we discarded an initial segment of the solution, which reflected the process of tending to the chaotic attractor from the given initial conditions, called transient, or relaxation, process. Therefore, the observed phase trajectory is very close to the attractor of the system, and in literature such phase portraits are often referred to as attractors for simplicity. The procedure of discarding the transients mimics a typical experimental situation, in which between the moment when the device is switched on and the moment when data registration starts, a noticeable amount of time elapses, and the system spontaneously converges to its attractor. In Figure S5**b** arrows show the reconstructed velocity vectors along the phase trajectory, obtained from numerical derivatives of all realizations with method (S6). Thus, this method permits an almost direct registration of the velocity vectors in an experiment, but only along the phase trajectory being recorded, i.e. only a tiny portion of the full velocity field can be obtained this way. It is impossible to experimentally register the velocity vectors outside this trajectory. The only way to obtain the full velocity vector field seems to be through detailed modelling from the first principles, as described in Section 1.1.

2.2. Case 2: a single realization is recorded

When observing the behavior of some spontaneously evolving device, more often than not the observer does not know how many state variables the respective dynamical system has. Moreover, often one can register only a single realization $a(t)$ characterising the behavior of the system, e.g. a time trace of voltage at a certain point of a neuron cell. This realization might coincide with one of the coordinates of the full phase trajectory $\mathbf{y}(t)$, e.g. be equal to $a(t)$=$y_1(t)$. However, under more general assumptions, it might be some scalar function f of all coordinates, $a(t) = f(y_1(t), y_2(t), \ldots, y_N(t))$, or some functional of the coordinates, usually reflecting the way the measurement instrument senses the system's behavior.

Assuming that there exists a dynamical model of the given device in the form (1)/(2), and that the observable behavior corresponds to an attractor of the system, in [58] a famous Takens's theorem was proved saying that one can reconstruct from $a(t)$ a set topologically equivalent to the original attractor under very generic conditions on the function f. To do this, one needs to perform a delay embedding procedure and construct a state vector $\mathbf{z}(t)$ whose components are the delayed versions of the same realization, namely,

$$\mathbf{z}(t) = (a(t), a(t-\tau), \ldots, a(t-(M-1)\tau)), \tag{S8}$$

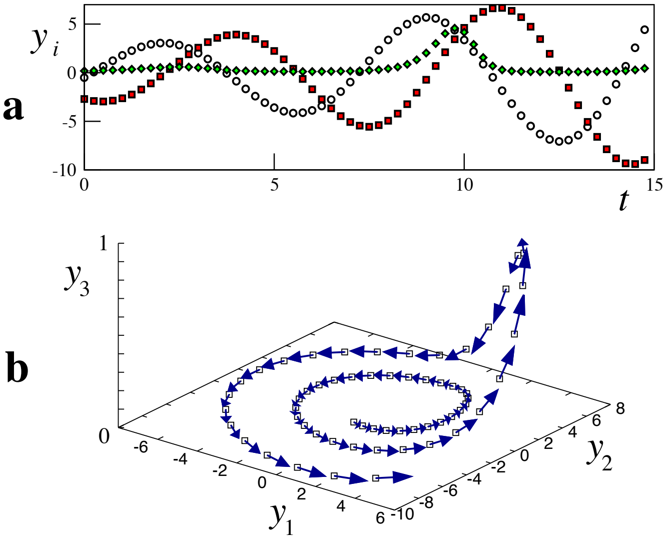

Figure S5. **a** Simulated "experimental data" representing state variables y_1 (white circles), y_2 (red squares) and $5y_3$ (green diamonds) of Rössler system (S7) sampled with step Δt. **b** Sampled phase trajectory $\boldsymbol{y}(k\Delta t)$ (squares) and the velocity vectors (arrows) estimated along this trajectory with method (S6). Namely, each arrow points in the direction of the velocity vector, and for the clarity of presentation the lengths of the arrows are equal to those of the respective velocity vectors divided by 5.

where τ is some time delay and M is an embedding dimension. Strictly speaking, M should satisfy $M>(2N+1)$ (or $M>(2d+1)$, where d is the dimension of the attractor[S12]), but in practice it is often chosen to be smaller, as demonstrated in[S13]. Some useful comments about this result can be found in[S14]. Although Takens's theorem states that the embedding according to (S8) will work with almost any value of τ, this statement is true only for infinitely long realizations, which are known exactly. However, in any real situation, the data are observed during only a finite time, are registered with some finite accuracy and, moreover, are affected by measurement noise. Several methods have been proposed to identify an optimal value of τ[S15,S16]. Another popular method to reconstruct a phase trajectory is the method of successive differentiation[S13],

$$z(t) = \left(a(t), \frac{\mathrm{d}a(t)}{\mathrm{d}t}, \frac{\mathrm{d}^2a(t)}{\mathrm{d}t^2}, \ldots, \frac{\mathrm{d}^{M-1}a(t)}{\mathrm{d}t^{M-1}}\right). \tag{S9}$$

In[S17] a method of successive integration has been proposed to deal with data comprising segments of very fast and very slow motion. A thorough overview of the theoretical foundations of the embedding methodology is given in[S18]. It has been noted that Takens's theorem applies only to noise-free data, but in later studies Takens's result was extended to noisy data as well[S19,S20].

One can combine different embedding methods to reconstruct the state vector $z(t)$. In Figure S6 several examples of attractor reconstruction are illustrated. Namely, in Figure S6**a** the original chaotic attractor of Rössler system (S7) is given with parameters indicated in the figure caption. Figure S6**b** illustrates attractor reconstruction from $a(t)=y_1(t)$ sampled with $\Delta t=0.1$ using delay embedding (S8) with $\tau=0.5$. The embedding dimension $M=3$ was chosen following[S13]. In Figure S6**c** we show an attractor reconstructed from $a(t)=y_1(t)$ with a combination of a delay (S8) with $\tau=0.5$ and a differentiation (S9) embeddings, namely, $z(t) = \left(a(t), a(t-\tau), \frac{\mathrm{d}a(t)}{\mathrm{d}t}\right)$. Finally, Figure S6**d** illustrates how an attractor can be reconstructed with exactly the same embedding parameters as in **b**, but from the realization $a(t)=y_1(t)+y_3(t)$. One can see that, as predicted by Takens's theorem, all attractors are topologically equivalent to each other. Also, the same embedding procedures can be used to reconstruct phase trajectories not only on the attractor, but also those representing transient dynamics[S21].

Sometimes in an experiment one can register several realizations, whose number would be less than the total number of state variables. Such would be the situation when in a circuit of coupled neurons the activity data (usually voltages) are measured from every neuron entering the network. One variable is certainly not sufficient to describe a single neuron realistically, hence the collection of such realizations would be insufficient for the reconstruction of the phase trajectory of the whole network. From the realizations available one can form a state vector, in which some components will be the recorded realizations, and the others their delayed versions or their derivatives. After the phase trajectory is reconstructed from experimental data, one can estimate the velocity vectors along this trajectory in the same manner as demonstrated in Section 2.1. State space reconstruction forms the basis for estimation of several important characteristics of the system dynamics, such as Lyapunov exponents[S22] and attractor dimension[S23], or estimation of the manifolds of fixed points[S24]. Methods of nonlinear time series analysis are well overviewed e.g. in a standard reference book[S25].

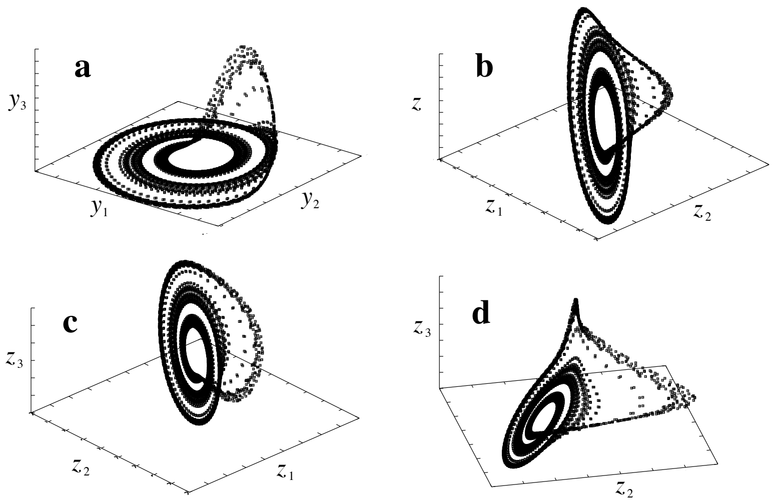

Figure S6. **a** Sampled phase trajectory of Rössler system (S7) at parameter values r=0.2, b=0.2 and c=4.62 after transients were discarded. This trajectory is very close to the chaotic attractor of this system and hence is often loosely called "an attractor". **b** Phase portrait reconstructed from a single realization $a(t)$=$y_1(t)$ of this system using method (S8) with $\tau = 0.5$. **c** Phase portrait reconstructed from a single realization $a(t)$=$y_1(t) + y_3(t)$ using (S8) with $\tau = 0.5$. **d** Phase portrait reconstructed from a single realization $a(t)$=$y_1(t)$ using a combination of (S8) with $\tau = 0.5$ and successive differentiation. All sets look topologically equivalent to each other, as predicted by Takens's theorem.

Although one cannot recover from the time series the *full* velocity field of the underlying system, there exist methods to reconstruct the velocity field from such data on the attractors[S26–S29], also overviewed in a dedicated book [39] and in[S25]. Perhaps counterintuitively, it has been shown that noise affecting the dynamics in a fairly general manner can help reconstruct the velocity field not only on the attractor, but in some vicinity of it[S30], because it forces the phase trajectory to visit the areas of the state space it would have not otherwise visited, and therefore to reveal the features of the velocity field in these areas in addition to those on the attractors.

3. Comparison of our Conceptual Model with State Space Augmentation

In the main text of the paper, we introduced into consideration a pair of Equations (7)–(8) as a conceptual model of a cognitive system. Equation (7) declares that the state $\boldsymbol{x}$ evolves according to the rules $\tilde{\boldsymbol{a}}$ that depend on $\boldsymbol{x}$, which is the usual definition of a dynamical system. At the same time, Equation (8) states that the rules $\tilde{\boldsymbol{a}}$ themselves evolve according to some other rules $\boldsymbol{c}$, which depend both on $\boldsymbol{x}$ and on the stimulus $\boldsymbol{\eta}(t)$, which is the new concept that we propose. Construction of the system (7)-(8) might superficially resemble the procedure of augmentation (or extension) of the state space of a dynamical system (7), in which the vector $\tilde{\boldsymbol{a}}$ is assumed to depend on parameters $\boldsymbol{p}$, which themselves change in time. In this case one can form an extended state vector $(\boldsymbol{x}, \boldsymbol{p})$ and an extended velocity field describing evolution rules for this new vector. However, system (7)–(8) cannot be obtained using the conventional state space augmentation, since our procedure is different from the latter both technically and conceptually, as explained below.

We start from the *technical* distinction. Within the conventional procedure of the state space augmentation used, for example, in control theory, one considers a dynamical system

$$\dot{\boldsymbol{x}} = \boldsymbol{f}(\boldsymbol{x}, \boldsymbol{p}(\boldsymbol{t}), \boldsymbol{\eta}(t)), \qquad \text{(S10)}$$

where $\boldsymbol{x} \in \mathbb{R}^N$ is a state vector of dimension N. With this, the right-hand side $\boldsymbol{f}$ in (S10) is a fixed deterministic function of both $\boldsymbol{x}$ and an M-dimensional vector of parameters $\boldsymbol{p}$, which is usually assumed to be changing with time t in order to adjust the behavior of $\boldsymbol{x}$ in a desired manner. In addition, $\boldsymbol{f}$ may depend on an external stimulus $\boldsymbol{\eta}(t)$. Thus, the system (S10) is non-autonomous. Often one wishes to construct an automatic control scheme, i.e. to prescribe the rules $\boldsymbol{g}$ according to which parameters $\boldsymbol{p}$ would evolve depending on the current state $\boldsymbol{x}(t)$ (see[S31] for an example)

$$\dot{\boldsymbol{p}} = \boldsymbol{g}(\boldsymbol{p}, \boldsymbol{x}). \qquad \text{(S11)}$$

As a result, one can introduce an augmented state vector $(\boldsymbol{x}, \boldsymbol{p})$ of dimension N+M, and combine (S10) and (S11) to obtain a

new dynamical system with the velocity field $(\boldsymbol{f}, \boldsymbol{g})$ describing its evolution, namely,

$$\dot{\boldsymbol{x}} = \boldsymbol{f}(\boldsymbol{x}, \boldsymbol{p}, \boldsymbol{\eta}(t)), \quad \dot{\boldsymbol{p}} = \boldsymbol{g}(\boldsymbol{p}, \boldsymbol{x}). \tag{S12}$$

Note, that (S12) retains the form of a standard dynamical system, either autonomous of the form (2) if there is no stimulus ($\boldsymbol{\eta}(t) \equiv \boldsymbol{0}$), or non-autonomous in the form (4) if the stimulus is present ($\boldsymbol{\eta}(t) \neq \boldsymbol{0}$). In dynamical systems, all state variables should be independent of each other, i.e. no variable can be a function of other variables. Thus, in (S12) one assumes that in the new state vector $(\boldsymbol{x}, \boldsymbol{p})$ all components are *independent*.

Suppose one wishes to interpret Equations (7)–(8) by analogy with (S12) and to consider a new "augmented" vector $(\boldsymbol{x}, \boldsymbol{a})$. Note, that it has to be $(\boldsymbol{x}, \boldsymbol{a}(\boldsymbol{x}))$, i.e. half of the variables would explicitly depend on the other half. Thus, unlike $(\boldsymbol{x}, \boldsymbol{p})$, the vector $(\boldsymbol{x}, \boldsymbol{a}(\boldsymbol{x}))$ cannot be a state vector, and one cannot construct a standard dynamical system of the form (2) describing its evolution. Also, the augmented system (S12) retains the standard form (2) or (4) of a dynamical system expressible as a set of ordinary differential equations (ODEs). On the contrary, (8) is principally a partial differential equation (PDE). Moreover, (8) is a special PDE which is difficult to interpret on its own because of the absence of a spatial derivative. Equation (8) acquires a clear meaning only if paired with (7).

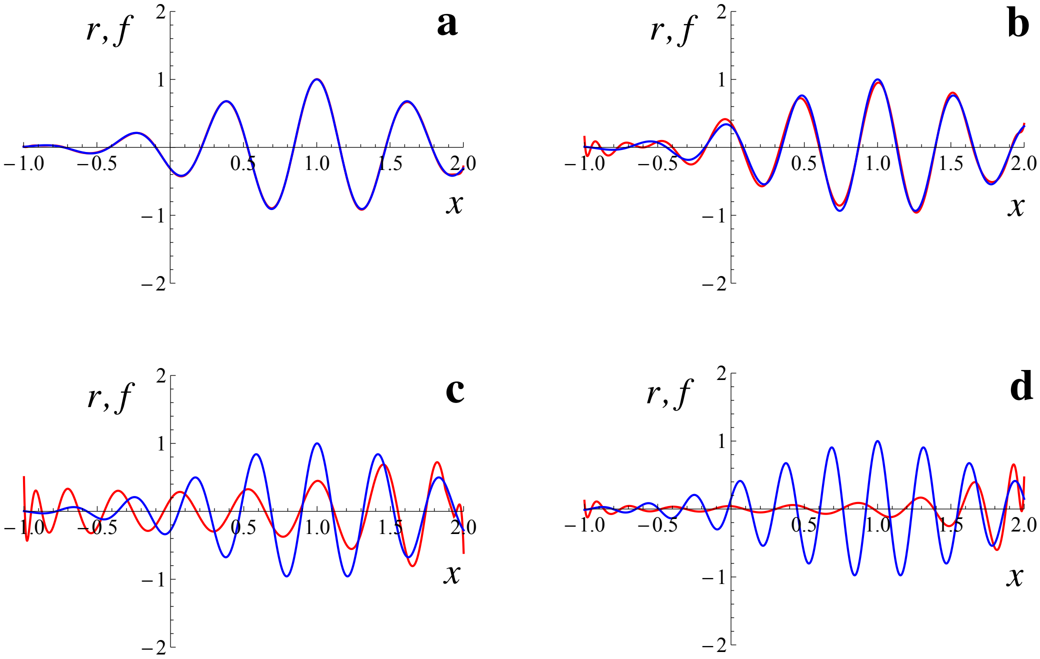

Figure S7. Illustration of the lack of plasticity in a parametrized function. Blue lines show the target scalar function r of a scalar argument x, $r(x) = \cos(n(x-1))\exp(-(x-1)^2)$ at different values of n: **a** n=10, **b** n=12, **c** n=15, and **d** n=20. Red lines represent a parametrized function $f(x)$, a polynomial of the 20th order, which in each case is chosen as the best fit of the target function. One can see that when the target function is relatively simple, the approximating function is almost indistinguishable from it (**a**). However, when the target function acquires a more complex shape by demonstrating more fluctuations per unit range of its argument, the approximating function starts to deviate from the target function (**b**). When the target function becomes too complex, the approximating function fails to reproduce not only its fine details, but also the general behavior (**c** and **d**).

Next, we explain the *conceptual* distinction between (S12) and (7)–(8). Conventional augmentation (S12) is possible only if the right-hand side of the system (S10) with $\boldsymbol{\eta} = \boldsymbol{0}$, i.e. of $\dot{\boldsymbol{x}} = \boldsymbol{f}(\boldsymbol{x}, \boldsymbol{p}, \boldsymbol{0})$, is given by the function $\boldsymbol{f}$ that can be parametrized with a *finite* number of parameters forming vector $\boldsymbol{p}$. An example of such an augmented system is the conventional neural network (5)-(6), whose state vector is $(\boldsymbol{x}, \boldsymbol{w})$. However, parametrized functions are fundamentally lacking plasticity. Namely, given some target function $\boldsymbol{r}(\boldsymbol{x})$ of an arbitrarily complex shape, it is generally impossible to find a value of $\boldsymbol{p}$ at which $\boldsymbol{f}$ would coincide with $\boldsymbol{r}$. This idea is illustrated with Figure S7 showing the scalar target function of a scalar argument, $r(x)$ (blue line), which has a relatively complex shape and makes several oscillations within a bounded range of its argument. Suppose we wish to describe $r(x)$ by some parametrized function $f(x)$, chosen to be a 20th-order polynomial in this example. The function $f(x)$

with the *finite* number of parameters cannot make more than a certain maximal number of fluctuations within the given range of its argument. Therefore, if the complexity of the target function grows, as shown in Figure S7 by going from **a** to **d**, starting from some level of complexity, the parametrized function fails to reproduce the features of the target function reliably with any choice of its parameters.

The goal of our paper is to create a conceptual, i.e. an idealized, model of a cognitive system, and we wish to eliminate any lack of plasticity in the right-hand side $\tilde{\boldsymbol{a}}$ of (7) that might be obscuring the phenomenon of our interest, namely, the spontaneous shaping of the velocity field under the influence of stimulus. We want $\tilde{\boldsymbol{a}}$ to be principally able to develop any number of minima and maxima within any range of $\boldsymbol{x}$, and generally to be able to take any smooth shape. For this reason we deliberately depart from parametrized velocity fields with limited plasticity, and in Eq. (7) assume that $\tilde{\boldsymbol{a}}$ is an infinitely plastic function of $\boldsymbol{x}$. Thus, Eq. (8) governs evolution of $\tilde{\boldsymbol{a}}$ directly, rather than through the parameters, and no smooth shape of $\tilde{\boldsymbol{a}}$ is forbidden this way.

4. Simulated Data

The performance of the one-dimensional dynamical system (9) and (14) is illustrated in Figure 3 using two numerically simulated examples, and function $g(z)$ was of the form determined by equation (11) with $\sigma_z = \sqrt{0.1}$. In both examples random signals $\eta(t)$ consisted of values from two different categories. However, in the first example (Figure 3 **a,b**) the successive values of input are statistically independent of each other (uncorrelated), while in the second example (Figure 3 **c,d**) the subsequent values statistically depend on what the previous values were (correlated).

For the first example we create signal $\eta(t)$ by taking Gaussian white noise $\xi(t)$ with zero mean and unit variance and applying a non-linear zero-memory transformation to it, i.e. $\eta(t) = F(\xi(t))$[S32]. Here F is a non-linear function, chosen in such a way that the probability density of $\eta(t)$ becomes $p_1^{\eta}(\eta)$=$0.01245\eta^4 + 0.1065\eta^2 + 0.0482$ with $\eta \in [-3, 3]$, whose negative is shown in Figure 3**a** by solid line at the front. The two peaks in the density describe two categories of input values, and the peak tops (well bottoms in Figure 3(a)) represent the most typical values from each category.

In the resultant signal $\eta(t)$, whose portion is shown in Figure 3**b** by filled circles, the consecutive values are *uncorrelated*. Evolution of the landscape $V(x,t)$ is illustrated in Figure 3**a** by a surface, and one can see that with time it converges to the density $p_1^{\eta}(x)$ taken with negative sign. Another illustration of the process of shaping is given in Figure 3**b** by the shades of the background, where the darker shade represents a deeper landscape. The landscape becomes instantaneously deeper at the spot where the new value of η appears, but progressively smoothes out.

In the second example, $\eta(t)$ is the numerical solution of the following stochastic differential equation

$$\frac{\mathrm{d}\eta}{\mathrm{d}t} = h(\eta) + 0.5\xi(t), \tag{S13}$$

where $\xi(t)$ is Gaussian white noise with zero mean and unit variance, and $h(\eta)$=$3(\eta - \eta^3)/5$. Eq. (S13) describes a particle moving in a double-well potential shaped as the negative of an integral of $h(\eta)$, under the assumption of a large viscosity, under the action of a stochastic force. The consecutive values of $\eta(t)$ are correlated [49] as illustrated in Figure 3**d** by filled circles: if at a certain time the input value is from one category, at the next time moment the input value is more likely to be from the same category, and the switches between different categories occur rarely. The negative of the density $p_1^{\eta}(x)$, estimated numerically from the realisation of $\eta(t)$ as a distribution histogram, is shown by a solid line in Figure 3**c**, and one can see that the landscape $V(x,t)$ approximately tends to this function as time goes by. The shaping of the same landscape is also illustrated in Figure 3**d**, where the depth of V is shown against the values of stimulus η.

One can see that eventually both landscapes in Figure 3 shape into the negatives of the respective densities, but if the stimulus values are uncorrelated, the convergence is faster.

References

S1. Harre, R. *The philosophies of science* (Oxford University Press, London, 1972).

S2. O'Connor, T. & Wong, H.Y. Emergent Properties, *The Stanford Encyclopedia of Philosophy* (ed. Zalta, E.N.), (Summer 2015 Edition) https://plato.stanford.edu/archives/sum2015/entries/properties-emergent/.

S3. Minati, G., Pessa, E. & Abram, M. (Eds) Systemics of Emergence: Research and Development (Springer–US, 2006).

S4. Goldstein, H., Poole, C. P. & Safko, J. L. Classical Mechanics (3rd ed.) (Addison-Wesley, 2001).

S5. Horowitz, P. & Hill, W. The art of electronics (Cambridge University Press, 1980).

S6. van der Pol, B. *Radio Rev.* **1**, 701, 704, 754 (1920).

S7. Voit, E.O., Martens, H.A. & Omholt, S.W. 150 Years of the Mass Action Law, *PLOS Comput. Biol.* **11**(1), e1004012 (2015).

S8. Zhabotinsky, A.M. A history of chemical oscillations and waves. *Chaos* **1**(4), 379–386 (1991).

S9. Hodgkin, A.L. & Huxley, A.F. A quantitatve description of membrane current and its applications to conduction and excitation in nerve. *J. Physiol.* **117**, 500-544 (1952).

S10. Hopfield, J.J. Neurons with graded response have collective computational properties like those of two-state neurons. *Proc. Natl. Acad. Sci. USA* **81**, 3088–3092 (1984).

S11. Rössler, O.E. An equation for continuous chaos. *Phys. Lett. A* **57**(5), 397–398 (1976).

S12. Takens, F. in *Nonlinear Control Systems Design* (eds. Huijberts, H.J.C., Nijmeijer, H., VanDerSchaft, A.J. & Scherpen, J.M.A.), 427–429 (Pergamon Press, Oxford, 1998).

S13. Packard, N. H., Crutchfield, J. P., Farmer, J. D. & Shaw, R. S., Geometry from a Time Series, *Phys. Rev. Lett.* **45**(9), 712–716 (1980).

S14. Sauer, T.D. Attractor reconstruction. *Scholarpedia* **1**(10), 1727 (2006).

S15. Fraser, A.M. & Swinney, H.L. Independent coordinates for strange attractors from mutual information. *Phys. Rev. A* **33**(2), 1134–1140 (1986).

S16. Liebert, W. & Schuster, H.G. Proper choice of the time delay for the analysis of chaotic time series. *Physics Letters A* **142**(2–3), 107–111 (1989).

S17. Janson, N.B., Pavlov, A.N. & Anishchenko, V.S. One Method for Restoring Inhomogeneous Attractors. *Int. J. of Bifurcat Chaos* **8**(4), 825–833 (1998).

S18. Sauer, T., Yorke, J. A. & Casdagli, M. Embedology. *J. Stat. Phys.* **65**, 579–616 (1991).

S19. Casdagli, M., Eubank, S. Farmer, J.D. & Gibson, J. State space reconstruction in the presence of noise. *Physica D* **51**(1–3), 52–98 (1991).

S20. Stark, J., Broomhead, D.S., Davies, M.E. & Huke, J. Takens embedding theorems for forced and stochastic systems, *Nonlinear Anal. Theory Methods Appl.* **30**(8), 5303–5314 (1997).

S21. Jánosi, I.M. & Tél, T. Time-series analysis of transient chaos. *Phys. Rev. E* **49**(4), 2756–2763 (1994).

S22. Wolf, A., Swift, J.B., Swinney, H.L. & Vastano, J.A. Determining Lyapunov Exponents from a Time Series, *Physica D* **16**, 285–317 (1985).

S23. Grassberger, P. & Procaccia, I. Characterization of Strange Attractors. *Phys. Rev. Lett.* **50**(5), 346–349 (1983).

S24. Ditto, W.L., Rauseo, S.N., & Spano, M.L. Experimental control of chaos. *Phys. Rev. Lett.* **65**(26), 3211–3214 (1990).

S25. Kantz, H. & Schreiber, T. Nonlinear time series analysis (Cambridge University Press, 1997).

S26. Cremers, J. & Hübler, A., Construction of Differential Equations from Experimental Data, *Zeitschrift für Naturforschung A* **42**(8), 797-802 (1987). doi:10.1515/zna-1987-0805

S27. Crutchfield, J.P. & McNamara, B.S. Equations of motion from a data series, *Complex systems* **1**, 417-452 (1987).

S28. Broomhead, D. S. & Lowe, D. Radial basis functions, multivariable functional interpolation and adaptive networks. *DTIC Document, Tech. Rep.* (1988).

S29. Baake, E., Baake, M., Bock, H. G. & Briggs, K. M. Fitting ordinary differential equations to chaotic data. *Phys. Rev. A* **45**(8), 5524–5529 (1992).

S30. Gradišek, J., Siegert, S., Friedrich, R. & Grabec, I. Analysis of time series from stochastic processes. *Phys Rev E* **62**, 3146–3155 (2000).

S31. Pyragas, K., Pyragas, V., Kiss, I.Z., & Hudson, J.L. Adaptive control of unknown unstable steady states of dynamical systems. *Phys Rev E* **70**(2), 026215 (2004).

S32. Stratonovich, R. Topics in the theory of random noise (Gordon and Breach, 1963).
